# *Trichomonas vaginalis* Cysteine Proteinases: Iron Response in Gene Expression and Proteolytic Activity

**DOI:** 10.1155/2015/946787

**Published:** 2015-05-18

**Authors:** Rossana Arroyo, Rosa Elena Cárdenas-Guerra, Elisa Elvira Figueroa-Angulo, Jonathan Puente-Rivera, Olga Zamudio-Prieto, Jaime Ortega-López

**Affiliations:** ^1^Departamento de Infectómica y Patogénesis Molecular, Centro de Investigación y de Estudios Avanzados del Instituto Politécnico Nacional (CINVESTAV-IPN), Avenida IPN 2508, Colonia San Pedro Zacatenco, 07360 México, DF, Mexico; ^2^Departamento de Biotecnología y Bioingeniería, Centro de Investigación y de Estudios Avanzados del Instituto Politécnico Nacional (CINVESTAV-IPN), Avenida IPN 2508, Colonia San Pedro Zacatenco, 07360 México, DF, Mexico

## Abstract

We focus on the iron response of *Trichomonas vaginalis* to gene family products such as the cysteine proteinases (CPs) involved in virulence properties. In particular, we examined the effect of iron on the gene expression regulation and function of cathepsin L-like and asparaginyl endopeptidase-like CPs as virulence factors. We addressed some important aspects about CPs genomic organization and we offer possible explanations to the fact that only few members of this large gene family are expressed at the RNA and protein levels and the way to control their proteolytic activity. We also summarized all known iron regulations of CPs at transcriptional, posttranscriptional, and posttranslational levels along with new insights into the possible epigenetic and miRNA processes.

## 1. Introduction

Almost all organisms require iron as a cofactor for many biochemical activities. Iron participates in all oxidation-reduction processes: that is, DNA synthesis, cellular detoxification, and oxygen transport [1]. To maintain an optimal balance, the cell tightly controls the intracellular levels of iron through “sensor” proteins that respond to changes in iron availability by transcriptional and posttranscriptional regulatory gene expression mechanisms [2, 3]. For many protist parasites iron is an essential nutrient for their survival in the host. Some of them have high-iron requirements (50–200 *μ*M) such as the amitochondriate protists,* Tritrichomonas*,* Trichomonas*,* Giardia*, and* Entamoeba* sp., surpassing those of the majority of eukaryotic and prokaryotic cells (0.4–4 *μ*M) [4].

The flagellated protist parasite* Trichomonas vaginalis* infects the urogenital tract and is responsible for human trichomoniasis, the most common nonviral sexually transmitted disease that has a strong impact on human health [5]. Trichomoniasis common symptoms include vaginitis, urethritis, and prostatitis and is associated with preterm delivery, low birth weight, pneumonia, increased infant mental retardation and mortality, and predisposition to HIV/AIDS infection and cervical and prostatic cancers. It is also responsible for pneumonia, bronchitis, and oral lesions in immunocompromised patients [6, 7].* T. vaginalis* develops a chronical infection under different urogenital microenvironments, mainly affecting women, showing that it is able to respond accordingly to the hostile environment during infection by modulating the trichomonal pathobiology as an adaptative response.

To study the genetic diversity in* T. vaginalis*, Conrad et al. [8] found 27 polymorphic markers (21 microsatellites and 6 single-copy genes) using different* T. vaginalis* isolates from diverse geographical origins. These authors demonstrated that these isolates have a high degree of diversity distributed only in four of the six chromosomes. Thus, the presence of two population types in trichomonad isolates, Type 1 and Type 2, was demonstrated worldwide. Type 1 isolates are located predominantly in Africa and Type 2 primarily in Mexico. The rest of the world has both types. The two types of trichomonad isolates show differences in the frequency of* T. vaginalis* virus infection by a double-stranded RNA virus (TVV) and metronidazole resistance. These differences could contribute to the ability of certain isolates to preferentially colonize the male urogenital tract in comparison with those found in the vagina. In addition to these, trichomonad isolates infected with TVV show different growth rates and virulence [9]. Furthermore, the presence of TVV has important implications in the disease pathogenesis and in the expression of trichomonad cysteine proteinases (CPs) [10].

In the urogenital tract,* T. vaginalis* is exposed to unfavorable conditions such as acidic pH, temperature, presence of lactobacilli, cyclic hormonal changes, epithelium desquamation, scarce nutrients, presence of zinc, fluctuation in polyamines and iron concentrations, menstrual blood flow, and other unknown factors. Thus, the parasite requires a great adaptive capacity to survive in this adverse environment.* T. vaginalis* modulates the expression of multiple virulence factors involved in cytoadherence, cytotoxicity, phagocytosis, hemolysis, immune evasion mechanisms, and induction of host cell apoptosis among others in order to survive, obtain nutrients, and maintain a chronic infection. Most of these properties and virulence factors are differentially regulated by iron [6, 7]. The regulatory effect of some environmental factors has been previously discussed in other reviews [7, 11, 12].

In this review, we describe the recent advances regarding the influence of iron on gene expression regulation and functions of cysteine proteinases as virulence factors and their endogenous inhibitors.

## 2. *Trichomonas vaginalis* and Iron

*T. vaginalis* has high requirements of exogenous iron (250–300 *μ*M). Iron is an essential element for its survival, metabolism, and multiplication in culture [13, 14]. Iron also regulates some of the trichomonal virulence properties by known and unknown mechanisms.* T. vaginalis* uses multiple sources of iron in the ferrous free form: lactoferrin (Lf), hemoglobin (Hb), and heme. It has multiple iron uptake systems. One of them is through a 136 kDa receptor for binding the host holo-Lf. Other receptors bind the cytochrome C or Hb and heme [7, 14–16], using the adhesins AP65 and AP51 [7, 16] as heme- and hemoglobin-binding proteins [17]. This parasite also internalizes ferritin, but not transferrin. Other important sources of iron are erythrocytes and epithelial cells. Two erythrocyte-binding proteins of 12.5 and 27.5 kDa help* T. vaginalis* to acquire iron from Hb [15].

The absence of iron in the culture medium reduces cell growth and induces morphological changes in* T. vaginalis* from ellipsoid or amoeboid to rounded parasites followed by flagella internalization and axostyle invagination by a mechanism not yet understood. These rounded and irregular parasite forms resemble the* Tritrichomonas foetus* pseudocysts, which were observed among parasites that underwent stress conditions, that is, cold and starvation [18, 19]. However, these forms are rarely observed among trichomonads grown in axenic cultures [19–21]. Thus, iron has an important role in the general physiology and morphology of* T. vaginalis.* In addition, morphological alterations are also accompanied by an extensive change in their protein profiles. In particular,* T. vaginalis* actin proteins are upregulated under iron-depleted conditions and may participate in the morphological changes just described [22]. These observations show that under different iron conditions both growth and protein synthesis are differentially regulated in trichomonads [20–22].

## 3. Cysteine Proteinases (CPs) in* T. vaginalis*: Classification, Structure, and Processing

### 3.1. Proteinases

Proteinases, also known as peptidases or proteases, hydrolyze the peptide bond in proteins and peptides. Proteinases are widely distributed and can be found in biological systems from viruses to mammals [23]. Proteinases account for monomers of 10 kDa to multimeric complexes of hundreds of kDa. These enzymes disrupt the peptide bond either within the polypeptide chain (endopeptidases) or at the amino or carboxy ends (exopeptidases). Based on the catalytic mechanism and the nature of the residue involved in hydrolysis, proteinases are classified as serine, threonine, aspartic, glutamic, metallo-, or cysteine proteases; however, other proteases with unknown mechanism also exist [24].

Cysteine proteinases (CPs) are subdivided into families according to the statistically significant sequence similarity among them and biochemical specificity to small peptide substrates. Families are grouped into larger clans (CA, CD, CE, CF, CL, CM, CN, CO, CP, CQ, and an “unassigned” clan) with a common ancestral progenitor. Members of different clans are not evolutionarily related. However, members of different families within a clan share a common ancestor [24].

This review will focus on CPs and their iron regulation at transcriptional, posttranscriptional, and posttranslational levels and role in the virulence of* T. vaginalis*. According to the draft of its genome,* T. vaginalis* has more than 400 proteinase-coding genes; whereas 220 correspond to the cysteine type, only 23 CPs have been detected by two-dimensional (2D) substrate gel electrophoresis (zymograms) [25, 26] that only correspond to nine different gene products after being identified by mass spectrometry [27]. These CPs are distributed into clans: CA, CD, CE, CF, CO, and CP [25].

Two main clans, CA and CD, are the most well known due to their high expression levels in the parasite and their identification by two-dimensional substrate gel electrophoresis and proteomics studies [26–31]. Clan CA is dubbed “papain-like” due to the high sequence homology with the* Carica papaya* proteinase. All* T. vaginalis* papain-like proteinases characterized to date belong to family C1 (cathepsin L-like). Clan CD is another important clan of CPs in* T. vaginalis*, particularly family C13 (legumain-like). All* T. vaginalis* cysteine proteinases characterized to date belong to families C1 and C13 (cathepsin L-like and legumain-like, resp.) [27–31].

#### 3.1.1. Papain-Like CPs

**Figure 1:**
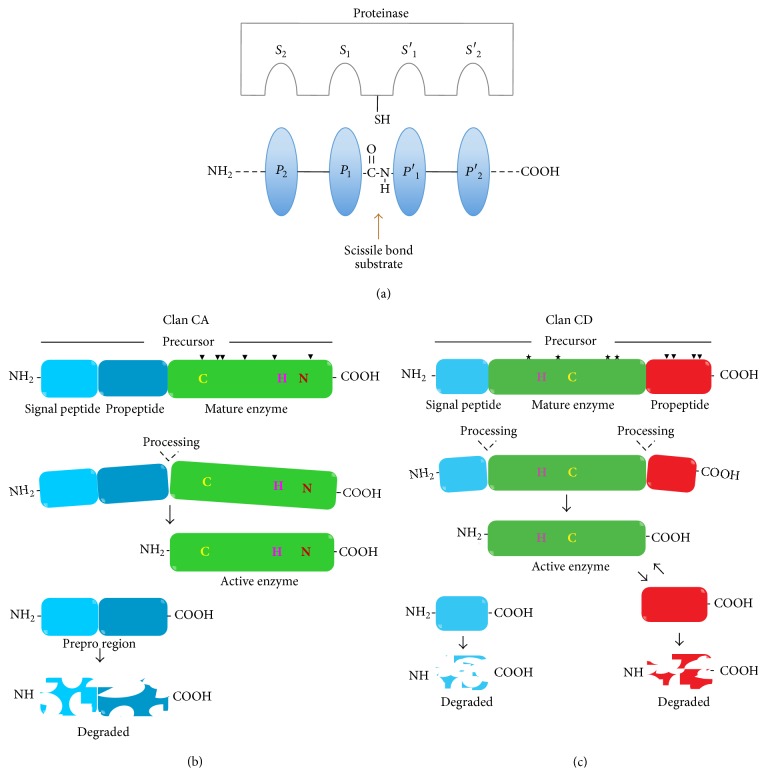
Processing involved in cysteine proteinase activation. (a) Representation of the interaction between substrate and the active sites of a cysteine proteinase. Subsites in the protease are denoted by “S” and subsites in the substrate by “P.” The active cysteine sulfhydryl nucleophile is represented as –SH and the scissile bond is shown. Processing of clan CA (b) and clan CD (c) cysteine proteinases; the signal peptide for cellular trafficking (light blue), the propeptide located in the N-terminus (dark blue) of the catalytic domain (green) in clan CA and in the C-terminus (red) of the catalytic domain (green) for clan CD. The catalytic residues C (Cys), H (His), and N (Asn) are indicated. Arrowheads show conserved Cys residues that can form disulfide bonds. Asterisks show N-glycosylation sites.

The catalytic site of papain-like CPs is highly conserved and formed by three residues denominated the catalytic triad: Cys25, His159, and Asn175 (papain numbering system). In the catalytic triad the Cys and the His residues form an ion pair stabilized by a hydrogen bond with Asn. The nucleophilic thiolate cysteine attacks the carbonyl carbon of the substrate and forms a tetrahedral intermediate, which transforms into an acyl enzyme with the simultaneous release of the C-terminal portion of the substrate. A water molecule hydrolyzes the acyl enzyme and a second tetrahedral intermediate is formed and cleaves into the free enzyme and the N-terminal portion of the substrate [31, 32]. The proteinase-binding region where the catalytic triad is located has binding pockets also known as subsites for the residues either side of the scissile bond in the substrate. Proteinase subsites in the N-terminal direction are named S_1_, S_2_, S_3_,…, S_*n*_, and subsites in the C-terminal direction are called S_1_′, S_2_′, S_3_′,…, S_*n*_′. In the substrate or inhibitor, the corresponding amino acids that bind to the subsites are named P_1_, P_2_, P_3_,…, P_*n*_ and P_1_′, P_2_′, P_3_′,…, P_*n*_′, respectively (Figure 1(a)). Members of the clan CA proteinases are either targeted to intracellular vesicle compartments such as lysosomes (cathepsin B, cathepsin L, and others) or are secreted if these possess a leader peptide. Clan CA proteinases are also sensitive to the irreversible inhibitor E-64 (L-trans-epoxysuccinyl-leucylamido(4-guanidino)butane) and have substrate specificity defined by the S_2_ pocket, in particular the amino acid residue 205 (papain numbering) [23]. For example, cathepsin L-like proteinases have alanine at this position, which cannot contribute to arginine binding; whereas cathepsin B-like proteinases have peptidyl-dipeptidase activity and an acidic group at this position that can preferentially bind to arginine (among any other residue), criteria used to distinguish them [23], using small peptides as substrates (Z-Phe-Arg-AMC and Z-Arg-Arg-AMC), where Z is an N-terminal blocking group, AMC is a fluorescent leaving group after hydrolysis. Mammalian cathepsin B can hydrolyse both substrates, whereas cathepsin L is limited to Z–FR–AMC only [23].

Nine* T. vaginalis* cathepsin L-like proteinases: TvCP1, TvCP2, TvCP3, TvCP4, TvCP4-like, TvCP12, TvCP25, TvCP39 (TvCPT), and TvCP65 have been partially characterized [7, 27, 33, 34] as virulence factors. All of them have a similar structure and motifs of cathepsin L-like CPs. In the parasite, family C1 members are expressed as zymogens, consisting of at least two regions: a prodomain and a catalytic domain (Figure 1(b)). In addition, TvCP2, TvCP3, and TvCP4 also have a signal peptide sequence [25, 35, 36]. Although structurally CA proteinases of* T. vaginalis* are cathepsin L-like, they possess substrate specificity that resembles cathepsin B and hydrolyze the fluorogenic substrates Z-FR-AMC and Z-RR-AMC (*K*_*m*_ values of 364 *μ*M and 160 *μ*M, resp.) [23].

**Figure 2:**
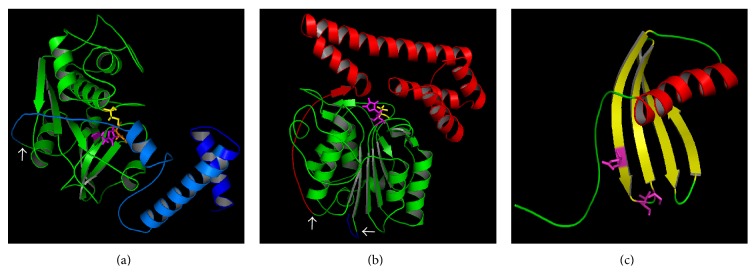
3D molecular model of TvCP4 and TvLEGU-1 precursors, and endogenous inhibitor TC-2 from* T. vaginalis*. (a) 3D model of the TvCP4 precursor showing the signal peptide (dark blue), the propeptide (light blue), and the catalytic domain (green). Catalytic residues Cys112 (yellow), His251 (magenta), and N271 (orange) are shown as sticks. (b) 3D model of the TvLEGU-1 precursor showing the signal peptide (light blue), the catalytic domain (green), and the propeptide (red). Catalytic residues His119 (magenta) and Cys164 (yellow) are shown as sticks. The arrows show the processing cleavage sites: TvCP4 (N84-A85) and TvLEGU-1 (C10-D11 and N260-E261). (c) TC-2 showing the amino acids: Gln, Val, and Gly of cystatin motif as magenta sticks. 3D models were obtained by using the I-TASSER server (http://zhanglab.ccmb.med.umich.edu/I-TASSER/) [39–41] and the models were visualized with the PyMOL Molecular Graphics System, Version 1.5.0.4 (Schrödinger, LLC, USA).

Apart from TvCP3, all cathepsin L-like CPs of* T. vaginalis* characterized up to date have six conserved cysteine residues (Cys_22_/Cys_63_, Cys_56_/Cys_95_, and Cys_153_/Cys_200_, papain numbering) forming three disulfide bonds and all of them possess the ERFNIN motif (EX_3_RX_2_[Ile/Val]FX_2_NX_3_IX_3_N), a characteristic of cathepsin L-like proteinases [25, 35]. Even though none of the crystal structures of the CPs of* T. vaginalis* have been elucidated, it is plausible they resemble that of the procathepsin L because the overall tridimensional fold of cathepsin L is highly conserved. The structure consists of two domains: L and R with the cysteine residue of the active site located in a structurally conserved *α*-helix of the L-domain where the histidine residue is in the R-domain. The propeptide occludes and runs in the opposite direction through the substrate binding cleft, which inhibits enzyme activity by sterically preventing the substrate from accessing the active site [37]. Recently, it was reported that the recombinant propeptide of the iron upregulated TvCP4 has a native-like conformation after* in vitro* refolding that works as an exogenous CP inhibitor of the proteolytic activity of certain* T. vaginalis* CPs from clan CA [38] (Figure 2(a)).

#### 3.1.2. Legumain-Like CPs

To date, two CPs of clan CD family C13 have been identified in* T. vaginalis*: TvLEGU-1 and TvLEGU-2 [42, 43]. Due to their similarity with legumain, a protease of legume* Canavalia ensiformis*, these CPs are called legumain-like. These CPs have a signal peptide, a propeptide, and a catalytic domain, but unlike clan CA CPs, the propeptide is located at the C-terminus of the catalytic domain [44]. For example, human legumain is synthesized as a zymogen and has various processing steps: a precursor (56 kDa), intermediate product (47 kDa), and active form (36 kDa). It is processed at an aspartic acid (D) at the N-terminal and asparagine (N) at the carboxyl terminus [45, 46] (Figure 1(c)). In legumains, the propeptide acts as a chaperone and stabilizes the catalytic domain at neutral pH. At acidic pH, the propeptide is cleaved as legumain goes through conformational rearrangements during activation [47]. The catalytic dyad in a clan CD member such as legumain-like CPs is His and Cys (Figure 1(c)) [48]. This type of proteinase possesses tightly defined substrate specificities at P1 position. CPs of family C13 of clan CD exclusively hydrolyse peptides and proteins on the carboxyl side of asparagine residues and are known as asparaginyl endopeptidases (AEPs). CPs of family C13 also show sequence similarity with glycosylphosphatidylinositol (GPI): protein transamidases. Therefore, these legumain-like proteinases can play a role in the attachment of GPI anchors to precursor proteins in the endoplasmic reticulum [23, 48]. Through an acyl transferase reaction, a peptide bond is formed between the terminal amine of the ethanolamide phosphate group of the GPI anchor and the C-terminal carbonyl group at the *ω* site of the protein [48]. Due to their strict substrate specificity, legumain-like proteinases are not inhibited by E-64. However, general thiol-blocking reagents such as iodoacetamide and iodoacetic acid can inhibit their proteolytic activity [23, 48]. Legumain selective inhibitors such as aza-peptide epoxides have been also discovered [49]. This characteristic together with the high immunogenicity of legumain-like CPs of* T. vaginalis* leads to proposing these CPs as prospects for drug design and as diagnostic tools [27, 43]. Recently, human and mouse legumain crystal structures have been elucidated, with an overall architecture of a central six-stranded *β*-sheet [*β*1–*β*6], flanked by five major *α*-helices (*α*1–*α*5) [46, 50]. The theoretical 3D model of TvLEGU-1 is shown in Figure 2(b).

## 4. *T. vaginalis* CPs Involved in Virulence Properties Are Differentially Modulated by Iron

The pathogenesis of* T. vaginalis* is a multifactorial process and its virulence is differentially modulated by iron [51]. In this parasite iron modulates both the expression of crucial metabolic enzymes and several virulence factors such as adhesins, a cell-detaching factor, and CPs, among other molecules, directly affecting virulence properties, accordingly [6, 7, 14, 52, 53].

Some CPs are differentially modulated by iron [33, 36, 54] and play crucial roles in certain virulence properties of* T. vaginalis*, including cytoadherence [7, 43, 52, 55], cytotoxicity [7, 34, 52, 56, 57], hemolysis [58–61], complement resistance [62], immune evasion [7, 52, 63], and induction of apoptosis in human cells [7, 52, 64–66] (Table 1). Virulence properties of* T. vaginalis* have been described in detail in recent reviews [7, 52, 67]. Moreover, trichomonad CPs are found in vaginal secretions of patients with trichomoniasis and some of them are immunogenic [7, 27, 52, 57, 68, 69]. Although the secretion pathway followed by CPs is still unknown in trichomonads, we could not discard that the presence of a signal peptide ensures the proteins to enter a secretory pathway via the endoplasmic reticulum as in any other eukaryote cell. Alderete and Provenzano [70] hypothesized that the* in vivo* synthesis of proteinases must somehow be under the control of environmental cues to modulate the number and amount of proteinases needed at any particular moment and microenvironmental condition during infection.

## 5. Only Few* T. vaginalis* CP-Encoding Genes Are Expressed: From the Genome to the Degradome of* T. vaginalis*

The publication of the draft of the* T. vaginalis* genome sequence was a breakthrough for research in this parasite, by providing an important platform for molecular and cellular studies. In a genome size comparison between* T. vaginalis* and other protist parasites it comes to light that this organism has one of the largest genomes with ~160 Mb spread in six haploid chromosomes [25]. The* T. vaginalis* genome sequence reveals that this parasite contains ~60,000 predicted protein-coding genes. At least ~65% of the genome sequence is repetitive and ~39 Mb corresponds to 59 repetitive families that can be classified as virus-like, transposon-like, retrotransposon-like, and other unclassified repetitive elements. Many gene families in* T. vaginalis* are represented by a high copy number. This conservative gene family expansion could facilitate the parasite adaptation to different environmental conditions. One of the largest families with ~880 genes corresponds to eukaryotic protein kinases (ePKs) and ~40 atypical protein kinases (aPKs), making it one of the largest eukaryotic kinomes known [24]. Several multigene families were found including some of the enzymes of the glycolytic pathway, cytoskeleton proteins, and Myb-like transcription factors with >400 genes [12].

**Figure 3:**
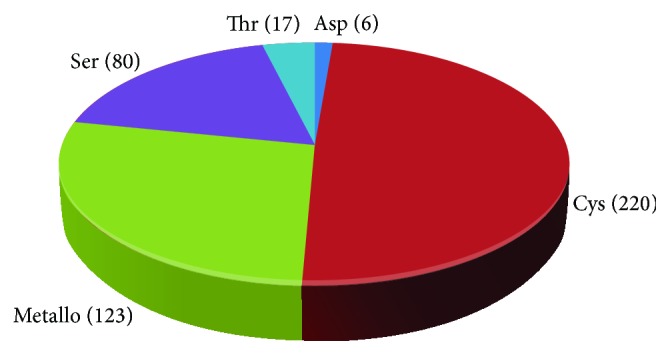
Classification of ~440 peptidase coding genes found in the* T. vaginalis* genome sequence database (http://www.trichdb.org/) as serine, threonine, aspartic, metallo-, or cysteine proteinase. The number of members per type of proteinases is shown in parenthesis: cysteine (220), metallo- (123), serine (80), threonine (17), and aspartic (6) [25].

*T. vaginalis* has ~440 peptidase-coding genes showing one of the most complex degradome described. This degradome includes proteolytic enzymes from different clans: aspartic AA (2), AD (4); cysteine, CA (185), CD (20), CE (9), CF (1), PC (C) (1), PB (C) (1), and U(-) (1); serine, SB (32), SC (36), SF (1), S- (9), and PC (S) (1); threonine, PB (T) (16), PB (T) (1); metallo-, MA (63), MC (11), ME (8), MG (13), MH (17), MK (1), and MP (7) (the number in parenthesis indicates the number of members in each clan) [25]. Half of the peptidase-coding genes (~220) are of the cysteine type (CPs), including ~48 members in family C1, which have sequences homologous to papain, and 10 members in family C13 of legumain-like CPs [25]. CPs are the major proteolytic enzymes expressed by this parasite (Figure 3).

*T. vaginalis* comparative transcriptomic analysis at large-scale gene expression level was performed as part of the collaborative work by several groups. It has generated an enormous collection of different expressed sequence tags (ESTs) from parasites cultured under defined conditions related to cell cycle, growth, iron depletion, restricted glucose starvation, cold, and pathogenesis. These data are available in the TrichDB genome sequence database (http://www.trichdb.org/) [25].

Taking into account this genomic approach and in view of the different transcriptome and proteome data generated from parasites grown under different iron concentrations, we conducted a compilation of the information about cysteine proteinases belonging to cathepsin L-like (Table 2) and legumain-like CP families differentially modulated by iron (Table 3). In this review we also analyzed the EST collection together with the results of several transcriptomes and proteomes recently published [27–31, 71]. We also included in these tables other important aspects related to information in the genomic context and the known function for each CP.

**Figure 4:**
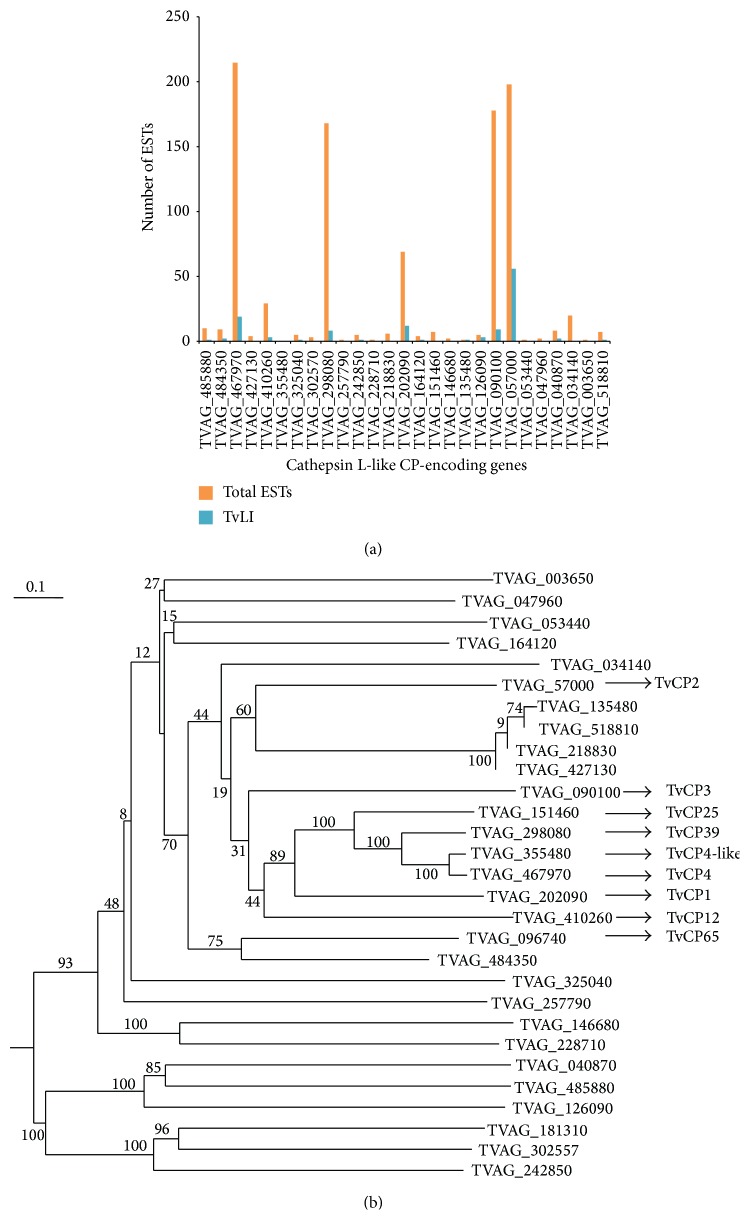
EST analysis and phylogenetic tree of cathepsin L-like CP-encoding genes expressed under different conditions. (a) Bar graph of the total ESTs (total EST, orange bars) found on the* T. vaginalis* genome database (http://www.trichdb.org/) compared with the number of ESTs expressed in parasites grown under iron-restricted conditions (TvLI, blue bars) (see Table 2). (b) Phylogenetic tree of expressed cathepsin L-like CPs using the DNAMAN program version 3.0 and a bootstrapping of 1000. The names of known cathepsin L-like CP-coding genes are shown.

**Figure 5:**
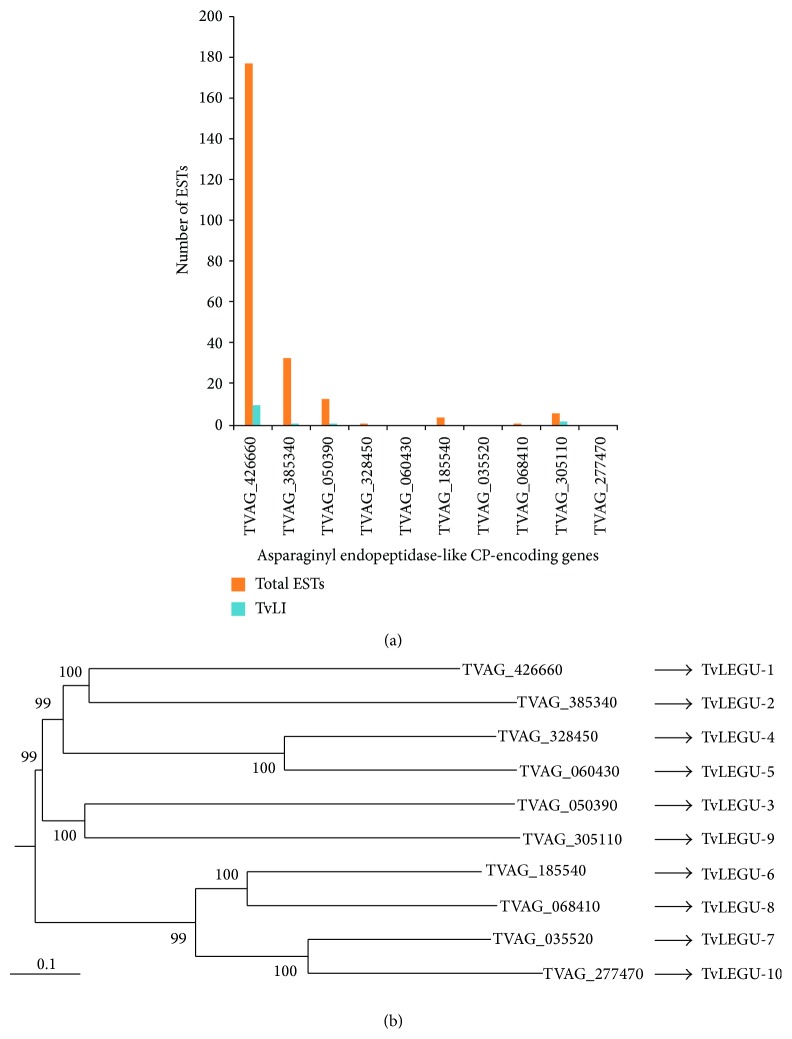
Total EST analysis and phylogenetic tree of asparaginyl endopeptidase-like (AEP-like) CP-encoding genes expressed under different conditions. (a) Bar graph of the total ESTs found on the* T. vaginalis* genome database (http://www.trichdb.org/) (Total EST, orange bars) compared with the number of ESTs expressed in parasites grown under iron-restricted conditions (TvLI, blue bars) (see Table 2). (b) Phylogenetic tree of legumain-like proteins using the DNAMAN program version 3.0 and a bootstrapping of 1000. The names of known AEP-like CP-coding genes are shown.

The transcript levels measured based on the number of existing ESTs in the genome database showed that only few CPs are being expressed at the mRNA level (Figures 4 and 5). These findings are in agreement with the results shown in the transcriptomic data [71] and also with the proteomics and functional studies [7, 27–31, 34, 43, 52, 57, 58]. In addition, phylogenetic analysis based on protein sequences of cathepsin L-like and legumain-like CPs revealed that in each group the CPs that are highly expressed in the EST analysis are clustered into closely related clades that appear to diverge from a common ancestor (Figures 4(b) and 5(b)).

At the protein level, the majority of the expressed proteins including the proteolytic enzymes of* T. vaginalis* are acidic, as predicted by a bioinformatics approach [28, 30, 31]. This may suggest that the presence of abundant acidic proteinases in the* T. vaginalis* proteome reflects an adaptation to the acidic microenvironment of the vagina that has a reducing environment, where the iron concentration is constantly changing throughout the menstrual cycle. These reducing conditions are sufficient for activation of trichomonad proteinases [70] given that the substrate degradation by many cysteine proteinases requires breakage of disulphide bonds under reducing conditions [26, 72–74].

**Figure 6:**
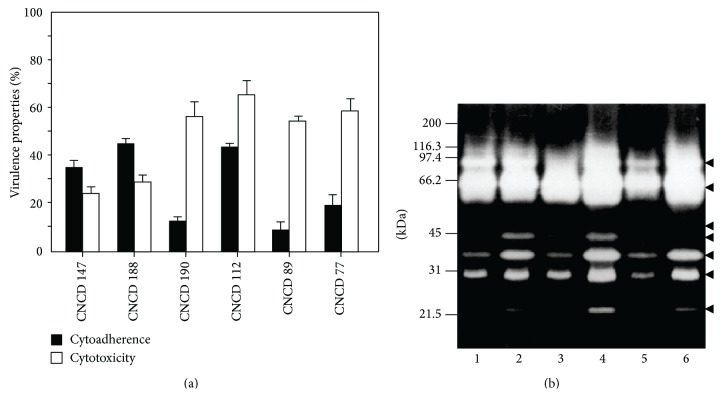
Cytoadherence and cytotoxicity levels in different* T. vaginalis* isolates and its differential proteolytical activity as zymogram profiles. (a) Percentage of the levels of cytoadherence and cytotoxicity of different fresh* T. vaginalis* isolates. Black bars: cytoadherence levels; white bars: cytotoxicity levels. (b) One-dimensional zymograms of protein extracts obtained from 4 × 10^4^ parasites from different fresh* T. vaginalis* isolates: lane 1: CNCD147; lane 2: CNCD188; lane 3: CNCD190; lane 4: CNCD 112; lane 5: CNCD89; and lane 6: CNCD77. Arrowheads show the positions of proteolytic activity as clear bands against a dark background. kDa: broad range molecular weight standards, (Bio-Rad) in kilodaltons (kDa).

Some CPs are more abundant in the amoeboid than in the ovoid form, suggesting that CP profiles of* T. vaginalis* isolates exhibiting high- and low-virulence phenotypes and differences in CP expression indicate that papain-like CPs are one of the key factors in cellular damage by* T. vaginalis* [28–30, 75]. The heterogeneity in peptidase expression could suggest that* T. vaginalis* strains are constituted by two phenotypically distinct subpopulations of parasites that would express qualitatively and/or quantitatively different proteins or enzymes involved in pathogenicity [29]. This was recently confirmed by a genomic analysis by Conrad et al. [8, 9]. Comparative analysis of the proteinase patterns in different trichomonad isolates with distinct levels of cytoadherence and cytotoxicity show heterogeneity in the proteolytic activity patterns (Figure 6).

Moreover, in the* T. vaginalis* genome sequence, 58 genes encoding papain-like and legumain-like CPs have been found [25], but only up to 23 spots with proteolytic activity between 23 and 110 kDa and p*I* between 4.5 and 7.0 have been detected in different isolates by 2D substrate gel electrophoresis (zymogram) [26, 27].

Remarkably, all the CPs identified to date, in spite of using distinct trichomonad isolates, growth conditions, and distinct forms of sample preparation, are almost the same in all cases. The information obtained has been based on the G3* T. vaginalis* genome sequence, showing that only few CP genes are being expressed in the different trichomonad isolates and strains analyzed [25, 26, 31, 33, 35, 42, 65, 68, 69]. Whether other CP genes have been expressed* in vivo* under other unknown environmental conditions found in the human genitourinary tract remains to be investigated.

Ramón-Luing et al. [27] showed that although* T. vaginalis* possesses an extremely complex degradome according to the genome sequence [25] only few CPs—seven cathepsin L-like CPs (TvCP1, TvCP2, TvCP3, TvCP4, TvCP4-like, TvCP12, and TvCP39) and two asparaginyl endopeptidase-like or legumain-like CPs (TvLEGU-1 and an uncharacterized AEP-like CP)—were identified in the active degradome of* T. vaginalis;* and some of these CPs have been characterized as virulence factors [7, 27, 52, 55–66].

For example, TvCP4, an iron upregulated CP, is a lysosomal and surface proteinase released* in vitro* by metabolically active parasites. It is a* T. vaginalis* virulence trait that plays a key role in hemolysis and expressed during infection. It can be considered as a potential biomarker for trichomoniasis [27, 58]. Like other genes in the* T. vaginalis* genome,* tvcp4* is a multicopy gene, and three TvCP4-like encoding genes have been reported [27, 35, 36, 65]. Although these CPs share high sequence identity (>96%), one is negatively regulated by iron and has been implicated in the induction of host cell apoptosis [65, 66], the iron upregulated TvCP4 is involved in hemolysis [58], and the iron regulation and function of the third TvCP4-like protein are still unknown. It appears to be a gene that is transcribed with an early stop codon, at least in the two* T. vaginalis* isolates from Mexican patients studied in our lab that may produce a smaller nonfunctional CP product (Lorenzo-Benito et al., our unpublished results) [27]. The presence of the three related* tvcp4* genes supports the hypothesis that the 48 genes coding for TvCPs belong to the cathepsin L-like group of the C1 family with genetic diversity, but with the same enzymatic active sites, conserved cysteine residues, and similar structural characteristics. In addition, these data suggest that all cathepsin L-like encoding genes in* T. vaginalis* may be the result of gene duplication and mutations derived from a single CP ancestor [76] as has also been shown in the phylogenetic analysis of expressed cathepsin L-like CPs (Figure 4(b)). Moreover, in the* T. vaginalis* degradome [27], TvCP4 was identified in five spots by 2D WB in the of 22 to 24 kDa region with different isoelectric points [58]. In this low molecular weight region is where most of the identified* T. vaginalis* CPs of clan CA have been found [27, 29, 65].

It is noteworthy to mention the detection of CP proteolytic activity in the ~60–65 kDa region that participates in cytotoxicity as TvCP65 [7, 52]. TvCP65 is downregulated by iron [56] and zinc and requires polyamines for its expression [7, 52]. TvCP65 is active at pH and temperature found in the vagina during infection and degrades proteins of the vaginal milieu such as collagen (Coll) IV and fibronectin (Fn). It is also located at the parasite surface and is immunogenic [7, 52]. However, in the genome of* T. vaginalis,* no genes were found encoding for active ~60–65 kDa cathepsin L-like CPs [25]. Interestingly, Ramón-Luing et al. [27] by a proteomic approach identified the protein spots from the 60–65 kDa region formed as a combination of at least two low molecular weight CPs (TvCP4 and TvCP2, or TvCP4-like with TvCP2, or even TvCP4 and TvCP4-like).

Although the characterization of TvCP2 is still in progress, by Western blot the anti-TvCP2 antibody detected a 65 kDa protein spot; by indirect immunofluorescence assays, TvCP2 and TvCP4 colocalized on the parasite surface (Lorenzo-Benito et al., our unpublished results). These results are consistent with the proteomic data reported by Ramón-Luing et al. [27] and support the association between these two CPs detected by MS, forming an active high molecular weight CP that participates in cytotoxicity as TvCP65 [7, 52, 56]. We can also speculate that a protein splicing mechanism [77] unheard of in these type of microorganisms could explain the association of two low molecular weight CPs to form a new higher size active CP species with new function such as TvCP65 [7, 52, 56]. Therefore, work needs to be done to identify TvCP2 function, iron regulation, and the mechanism involved in CP-complex formation between CPs and the environmental conditions that trigger it.

TvCP39 is another proteinase of the cytotoxic surface proteinases that interacts with the surface of HeLa cells and is also downregulated by iron [34, 57] and zinc [7, 52] and requires polyamines for its expression and nuclear localization [78]. TvCP39 was identified as part of the* T. vaginalis* active degradome [27]. TvCP39 is detected as a single proteolytic spot of ~39 kDa and p*I* 4.5 in 2D substrate gel electrophoresis. It was identified by proteomic and mass spectrometry (MS) in several protein spots with different sizes (45, 37.5, 28, 27, and 24 kDa). However, this CP is encoded by a <1000 bp gene for a 34 kDa precursor cathepsin L-like CP. TvCP39 is glycosylated, degrades several extracellular matrix proteins (fibronectin, distinct types of collagen), immunoglobulin G (IgG), and IgA, and hemoglobin, is immunogenic, and can be found in vaginal secretions of patients with trichomoniasis. It has proteolytic activity at 37°C in a broad pH range, similar to the conditions found during infection in women and men [7, 34, 52, 57]. Interestingly, Sommer et al. [65] also found this peptidase as part of the secreted CPs of the 30 kDa region. It was named CPT (TvCPT) and was implicated in the induction of host cell apoptosis together with other CPs of the  ~30 kDa region that are secreted by* T. vaginalis* grown under iron-restricted conditions [27, 65, 66]. The genomic sequence helped to clarify that TvCP39 and TvCPT correspond to the same molecule that is encoded by a unique gene,* tvcp39*, with high identity to TvCP4; both were part of the secreted CPs that could cause cellular damage by inducing programmed cell death [57, 65].

**Figure 7:**
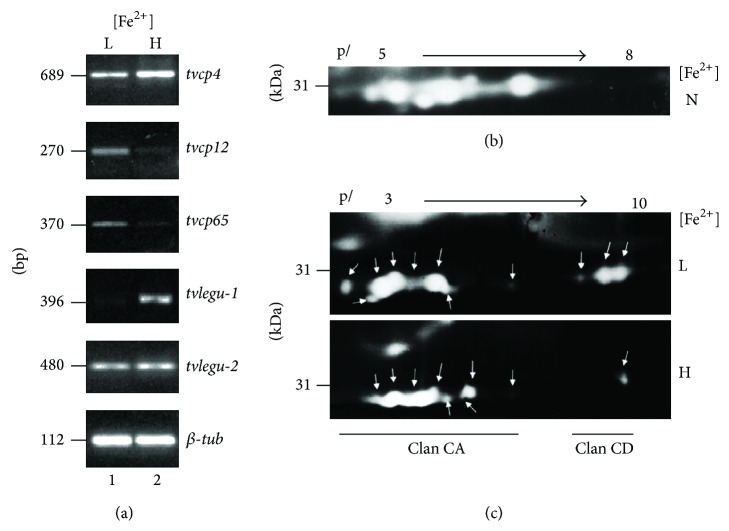
Effect of iron on the expression and proteolytic activity of* T. vaginalis* proteinases. (a) Semiquantitative RT-PCR using cDNA from parasites grown under iron-depleted (L) and iron-rich conditions (H) using specific primers to amplify several cathepsin L-like CP genes (*tvcp4*,* tvcp65*, and* tvcp12*) and AEP-like (*tvlegu-1* and* tvlegu-2)* CP genes. The*β-tubulin* gene was used as a loading control. (b) 2D zymograms of the 30 kDa region parasite proteinases obtained from trichomonads grown in normal iron conditions (N), (c) iron-depleted (L), and iron-rich conditions (H) separated over p*I* range 3–10. The arrows show the proteolytic spots of CPs from clan CA and clan CD that show differences depending on the iron conditions [7, 36, 41–43, 52].

Some of the CPs of the ~30 kDa region bind to the surface of HeLa cells and are necessary for cytoadherence (TvCP30) [7, 52]. This region is formed by at least six spots with proteolytic activity that corresponds to two distinct CP families: the papain-like family of clan CA, represented by four spots with p*I* between 4.5 and 5.5, and the legumain-like family of clan CD, represented by two spots with p*I* 6.3 and 6.5 [42] differentially regulated by iron at the transcript and proteolytic activity levels (Figures 7(b) and 7(c)) [54]. The family C13 of peptidases includes two distinct groups with different functions, the glycosylphosphatidylinositol (GPI): protein transamidase and the asparaginyl endopeptidase (AEP). Interestingly, TvLEGU-1 and TvLEGU-2 share ~30% amino acid identity with AEPs and ~26% with the GPI: protein transamidases [42]. We also showed that the amount of TvLEGU-1 transcript is positively regulated by iron, whereas the TvLEGU-2 mRNA is not affected by it [54] (Figure 7(a)). Of the ten legumain-like proteinases described in the* T. vaginalis* genome sequence [25], TvLEGU-1 [42] has been characterized at the functional level, playing a key role in trichomonal cytoadherence, and is located in lysosomes and Golgi complex and at the parasite surface in the presence of iron [43]. It also showed different levels of phosphorylation [43] and glycosylation (Rendón-Gandarilla et al., our unpublished results). Moreover, it is one of the most immunogenic CPs in patients with trichomoniasis and is detected in vaginal secretions during trichomonal infection [27, 43]. These data suggest that, during infection,* T. vaginalis* responds to different iron concentrations by differentially modulating the expression of several CPs [54], such as TvCP4, TvCP39, TvCP65, and TvLEGU-1 [7, 52]. Thus, it is reasonable to consider that both survival and the establishment of an infection in the host will depend on the ability of* T. vaginalis* to adapt to such environmental changes, including variations in host iron levels.

Furthermore, another important point to emphasize is some discrepancies between the theoretical and experimental p*I* and MW found in different proteins reported in proteomes, including CPs. These disparities could be explained as part of the processing steps during the maturation of the precursor CPs to remove the signal sequence and the N-terminal or C-terminal domain of cathepsin L-like or legumain-like, respectively, necessary for CPs activation [23–32, 48] as observed with the lower-size protein spots identified by MS as part of TvCP4 and TvCP39 [57, 58]. The differential posttranslational modification such as phosphorylation and glycosylation could also contribute to changes in p*I* and size as in TvLEGU-1 and TvCP39 [43, 58]. We can also speculate that the differences between experimental and predicted CP molecular masses could be due to unknown mechanisms for this parasite such as protein splicing [77]. Thus, TvCP65 is possibly formed by the combination of two lower-size CPs [27].

## 6. Molecular Mechanisms Involved in Gene Expression Regulation of CPs by Iron

To understand why few CPs are expressed at the mRNA and protein levels, in spite of the large number of genes encoding CPs as part of the extensive* T. vaginalis* degradome [25], it is necessary to review the possible molecular mechanisms involved in gene expression regulation at different levels. These mechanisms may include regulation at the transcriptional, posttranscriptional, and posttranslational levels or even some unexplored mechanisms that may include regulation by microRNAs (miRNA) and epigenetic mechanisms [7].

### 6.1. CP Regulation at the Transcriptional Level

The information about the mechanisms involved in gene expression regulation at the transcriptional level for CPs or other genes in* T. vaginalis* is limited. A stringent differential transcription regulation is suggested by the EST analysis carried on different types of genes, including housekeeping genes [25, 79].

**Figure 8:**
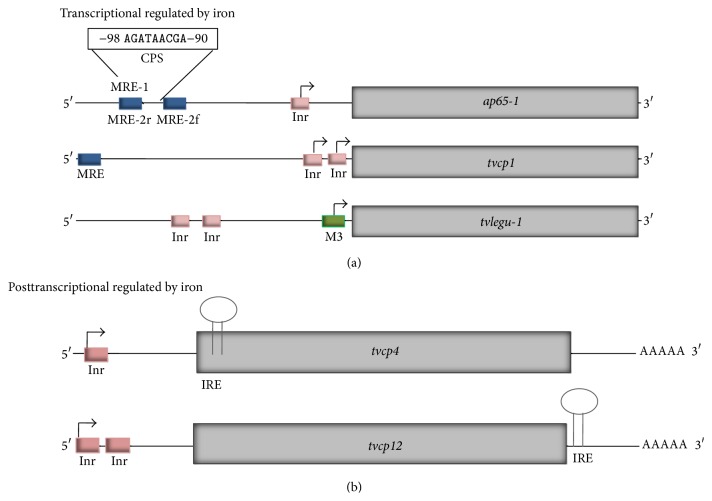
Genomic structure of different CP genes regulated by iron at the transcriptional or posttranscriptional level. (a) Transcriptional level. Comparison between the previously described iron inducible* ap65-1* gene promoter [85–87] and other two genes regulated by iron: the* tvcp1* gene promoter that responds to low-iron concentration. The* tvlegu-1* gene that responds to high-iron condition has a Motif 3 (M3, green box) element in the 5′-region, where the transcription start site (TSS) is also found (Figure 9). CPS: iron responsive core promoter sequence, MRE (blue boxes), Myb recognition element; Inr (pink boxes), initiator element; arrow: TSS. (b) Posttranscriptional level: comparison between two different genes with IRE hairpin loop elements located in the 5′-UTR,* tvcp4* mRNA, or in the 3′ UTR,* tvcp12* mRNA where the RNA-binding protein will bind under low-iron concentrations.

The identification of the transcription start sites (TSS) of several trichomonad genes, including those encoding virulence factors, shows a highly conserved sequence surrounding the TSS with a consensus sequence T C A + 1 Py (T/A) that is similar to the metazoan initiator-like element (Inr, Motif 1) [80, 81] that function as an alternative core promoter element for gene transcription in some organisms. This sequence is present in ~75% of the genes in the* T. vaginalis* genome sequence. It is recognized by transcription factors associated with the RNA polymerase II (RNApol II) and is responsible for TSS selection [25, 81, 82]. The IBP39, a 39 kDa Inr-binding protein, recognizes the Inr element and binds the transcriptional factor IID (TFIID) and the RNApol II to initiate the transcription. The interaction between the IBP39 and the Inr sequence is depending on the presence of certain conserved nucleotides [83]. IBP39 has been crystallized and characterized as an Inr-binding protein [83, 84]. Moreover, at least 100 proteins with the identified Inr-binding domain and characteristics similar to IBP39 have also been found [81]. An* in silico* analysis shows that several CP genes have this specific motif in the upstream region. In many cases, CP genes have one or two Inr elements. The distal element is usually the functional one [80, 81]. For example, primer extension and 5′-RACE analyses of the* tvcp12* mRNA from parasites grown under different iron concentrations show that the distal TSS is the functional one also for this CP (Figure 8(b)) (León-Sicairos et al., 2015, under revision).

Moreover, the transcriptional regulation mediated by iron has been described only for the* ap65-1* gene (Figure 8(a)). This gene encodes the AP65 adhesin, a 65 kDa surface protein involved in cytoadherence with sequence homology to a malic enzyme [6, 7, 16]. The* ap65-1* gene contains an iron responsive promoter that includes a core promoter sequence with a single Inr and eight closely spaced regulatory elements including three Myb (a DNA-binding protein that functions as a transcription factor first identified in myeloblastosis) recognition elements (MRE): MRE-1/MRE-2r and MRE2f [85–87] (Figure 8(a)). These sequences are recognized by three Myb-like transcription factors in* T. vaginalis*, TvMyb1, TvMyb2, and TvMyb3, and are responsible for the iron regulation of the* ap65-1* gene expression. These three proteins are responsible for the basal and iron-inducible transcription regulation through their interaction with MRE sequences. One of the most important features of this type of regulation is the Myb3 phosphorylation and nucleus translocation in response to iron concentration; please see below [88, 89].

The* T. vaginalis* genome contains ~400 Myb protein-encoding genes sharing 40–52% similarity. The three amino acids essential for DNA binding to the MRE sequences are present in all Myb-like proteins. Interestingly, by an* in silico* analysis, several MRE-like sequences have been identified in the 5′-region of genes encoding virulence factors. These MRE-like sequences can be recognized by various Myb proteins in response to iron or other physiological conditions, providing a higher plasticity in this type of regulation [81, 85–89].

Moreover, searching for the iron responsive promoter including the MRE-like elements in transcriptionally iron up- or downregulated CP genes revealed that none of them have all the regulatory elements identified in the* ap65-1* gene, but two CP genes have at least the eukaryotic MRE consensus sequence (C/T]AACG[G/T). One of these genes encodes for an unknown cathepsin L-, S-, or H-like CP TVAG_242850. Only 5 EST sequences were found for this gene; one of them is from low-iron condition library (Table 2). The other gene corresponds to the CP1 protein previously described by Mallinson et al. [35]. This protein is overexpressed under low-iron condition (Figure 8(a), Table 2). Thus, further work is required to solve whether these MRE-like motifs participate in a new transcriptional iron regulation mechanism using different Myb-like proteins, in addition to the one already described for* ap65-1* [85–89].

**Figure 9:**
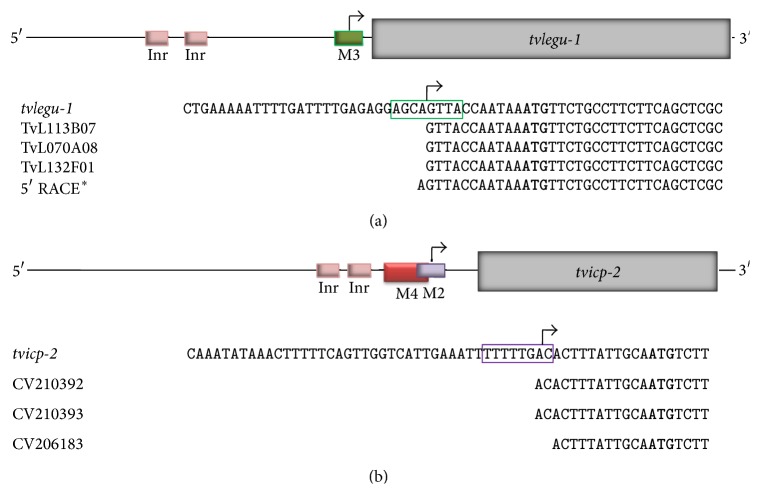
*tvlegu-1* and* tvicp-2* transcription start site identification. (a) Genomic organization compared with the ESTs (http://www.trichdb.org/) and 5′-RACE analysis of* tvlegu-1* mRNA to identify the possible TSS [42], Rendón-Gandarilla unpublished data. (b) Genomic organization of* tvicp-2* gene showing the putative Motif 4 (M4, red box) and Motif 2 (M2, purple box) compared with the ESTs (http://www.trichdb.org/) of* tvicp-2* mRNAs in different growth conditions to identify the possible TSS [90]. Motif 3 (M3, green box). Arrow: TSS.

By an* in silico* analysis in search for alternative basal promoter sequences to the Inr motif 1 (M1), overrepresented motifs located at the 5′-region of some* T. vaginalis* genes were found and grouped into four additional motifs (M2, M3, M4, and M5). Motif 3 resembles the metazoan MRE element and is recognized by the nuclear protein M3BP, a Myb-like protein, and Motif 5 is reminiscent of the Inr element [81, 82]. Interestingly, the TvLEGU-1-coding gene that is upregulated by iron lacks the iron responsive promoter elements described for* ap65-1.* Instead, it has two putative Inr sequence and a Motif 3 (M3) (Figure 8(a)). 5′-RACE experiments using RNA from parasites grown in different iron concentrations show that none of the Inr sequences were used as transcriptional start site; instead, it was found in the M3 sequence. The EST analysis confirmed these results (Figure 9(a)) (Rendón-Gandarilla et al., our unpublished data). Therefore, we found genes that in spite of having Inr sequences used alternative motifs as promoters. More work is needed to determine whether these motifs participate in iron regulation.

### 6.2. Posttranscriptional Regulation for CPs

Almost all organisms use iron as a cofactor for multiple biochemical activities. However, an excess of iron produces oxidative stress. To control intracellular iron levels and prevent its toxic effects, in vertebrates the iron homeostasis is regulated at the posttranscriptional level mediated by an IRE/IRP system. This mechanism is based on RNA-protein interactions between iron regulatory cytoplasmic proteins (IRPs) and stem-loop structures or iron responsive elements (IRE) located at the untranslated regions (UTRs) of certain iron-regulated mRNA [1, 91]. These RNA-protein interactions only occur under low-iron conditions. There are two possible scenarios depending on the location of the IRE element. (1) For genes that are upregulated by iron, that is, ferritin (FER), which is an iron-storage protein, in its mRNA, the IRE element is located at the 5′-UTR (IRE-*fer*). Under low-iron concentrations, IRP-1 and IRP-2 bind to the IRE-*fer* RNA, inhibiting its translation. In high-iron concentrations the IRP-1 is a multifunctional protein that acquires an aconitase activity instead, whereas IRP-2 is degraded; thus, the translation complex recognizes the mRNA and it is translated into the FER protein. (2) For genes that are downregulated by iron, that is, the transferrin receptor (TFR), in its mRNA, the IRE element is located at the 3′-UTR (IRE-*tfr*). Under low-iron conditions IRPs bind to the IRE-*tfr* RNA, preventing its degradation and increasing the half-life of the mRNA and the amount of translated TFR protein. In contrast, under high-iron concentrations, IRPs cannot bind to the IRE-*tfr* hairpin loops, the mRNA is degraded, and no TFR protein is synthesized [91].

Although the IRE/IRP system is a conserved iron regulatory mechanism throughout the evolution,* T. vaginalis* lacks aconitase activity and genes that encode for aconitase or IRP-like proteins. However, it has genes that are differentially regulated by iron at the posttranscriptional level such as those that encode for TvCP4 and TvCP12 CPs. The mRNA of these CPs contain a hairpin-loop structure at the 5′-UTR (*tvcp4)* or at the 3′-UTR (*tvcp12)*, respectively [36, 54] (Figure 8(b)). The RNA hairpin structures specifically bind to human IRPs [36] and to proteins present in cytoplasmic extracts from* T. vaginalis* grown under iron-restricted conditions [92]. Analysis of the* T. vaginalis* genome sequence revels that this parasite lacks genes coding for proteins with homology to the typical mammalian IRPs. Therefore, this parasite has an iron regulatory mechanism mediated by RNA-protein interactions that is parallel to the typical IRE-IRP system. The RNA-protein complexes are formed between atypical RNA IRE hairpin structures and multifunctional cytoplasmic proteins [54]. Recently, Calla-Choque et al. [92] reported the presence of four trichomonad cytoplasmic proteins that specifically bind to the IRE-*tvcp4* RNA. One of these proteins was identified by MS as the* T. vaginalisα*-actinin3 (TvACTN3) and characterized as an RNA-binding protein that could be involved in the iron posttranscriptional regulation in* T. vaginalis*. Functional assays demonstrate that this protein specifically interacts with the human IRE-*fer* and the trichomonad IRE-*tvcp4* RNAs [92].

### 6.3. CP Regulation at the Posttranslational Level by Iron

In addition to gene expression of CPs by a transcriptional or posttranscriptional regulation, other mechanisms are being studied to understand how the function of these proteins is regulated after being translated. The posttranslational regulation is frequently mediated by protein-protein interactions or protein modifications.

#### 6.3.1. Cystatins in* T. vaginalis* (Trichocystatins)

Trichocystatins are endogenous inhibitors of CPs in* T. vaginalis* that may participate in the posttranslational iron regulation mediated by protein-protein interactions. The best characterized inhibitors of cathepsin L are cystatins that belong to the MEROPS family I25 (clan IH) [93, 94]. Their members have structural and functional similarities and are classified into three main subfamilies: stefins, cystatins, and kininogens. The stefins (Type 1, subfamily I25A) are intracellular nonglycosylated single chain proteins (~11 kDa) and highly stable in a wide pH range [95]. The cystatins (Type 2, subfamily I25B) are extracellular proteins (~13 kDa) synthesized with a signal peptide [96], are nonglycosylated, and have two C-terminal disulfide bonds [93]. The kininogens (Type 3, subfamily I25C) are the largest CP inhibitors. They consist of an N-terminal heavy chain and a C-terminal light chain linked by a disulfide bridge with three tandemly repeated cystatin-like domains (D1, D2, and D3) [97]. All of them are potent, reversible, and competitive inhibitors acting in intracellular compartments and in the extracellular environment [98].

The principal function of cystatins is the protection of the cell from undesirable proteolysis [99]. The cystatins have been found in nematodes, platyhelminths, bacterial pathogens [100], and arthropods unlike parasitic protozoa where the cysteine protease inhibitors (ICPs) are commonly found instead [101], except for* Acanthamoeba* that has a cystatin-like inhibitor [102] involved in encystations and* T. vaginalis* that has three endogenous cystatin-like inhibitors, trichocystatins [25]. Interestingly, cystatins in parasites not only have the characteristic domains (a G domain in the N-terminal, the reactive Q × V × G domain in the central region, and the hairpin loop PW domain in the C-terminal domain) [98] necessary for inhibitory activity, but these inhibitors also perform a wide variety of specific functions as part of their biology.

In* T. vaginalis,* three genes encoding cystatin-like endogenous CP inhibitors, trichocystatins (TC-1, TC-2, and TC-3), have been identified in its genome sequence [25]. In the* T. vaginalis* active degradome the trichocystatin-2 (TC-2) inhibitor was identified by MS together with TvCP39 in a 45 kDa protein spot [90]. TC-2 belongs to the stefin subfamily of the cystatin family I25, is located in the cytoplasm and lysosomes of the parasite, and inhibits the proteolytic activity of papain, cathepsin L, and some of the cathepsin L-like CPs of trichomonads mainly TvCP39 and TvCP65 as observed in the zymograms [90].

Trichocystatin-2 (TC-2) plays a key role in regulating the TvCP39 proteolytic activity affecting trichomonal cytotoxicity [90]. TvCP39 has been characterized as a virulence factor cytotoxic to the target cell [7, 34, 52, 57]. The gene expression regulation of this CP by iron and polyamines has been investigated [7, 34, 78]. Its regulation by the endogenous CP inhibitor TC-2 is under investigation (Puente-Rivera et al., 2015, under revision). TvCP39 and TC-2 are associated and colocalized in some cytoplasmic vesicles, possibly lysosomes, suggesting* in vivo* regulation through specific protein-protein interactions. Pretreatment of live parasites with recombinant TC-2 reduced the levels of the trichomonal cytotoxicity towards HeLa cells in a concentration-dependent manner [90]. Iron upregulates the expression of this inhibitor and its target CP at the transcript and protein levels and the complex formation with several CPs (Puente-Rivera et al., 2015, under revision). Thus, these protein-protein interactions between TC-2 and its target CPs could be one of the posttranslational regulatory mechanisms in trichomonads that may contribute to protecting the parasite from the unwanted CP proteolytic activity. However, we could not ignore the hypothesis that this CP inhibitor could also have a particular function in the host cells during the host-parasite interplay.

Trichocystatin-3, TC-3, is also being studied in* T. vaginalis* isolates from different phylogenetic groups (Type 1 and Type 2) [9]. Its expression appears to be downregulated by iron, an opposite behavior to TC-2. TC-3 expression under iron-restricted conditions is more prominent in Type 2 than in Type 1 isolates (Sánchez et al., our unpublished data).

The presence of three endogenous CP inhibitors in* T. vaginalis* and the expression of at least two of them (TC-2 and TC-3) may be another level of regulation, in addition to those described so far in* T. vaginalis.* However, we could not exclude that this parasite could use some of the mechanisms already described for other pathogens to carry out the successful parasitism to the host because some virulence properties are shared among pathogens and some of these functions are regulated by iron. The interaction of CP/cystatin can stimulate some of these functions as in* Streptococcus pyogenes* where the CP IdeS/cystatin C complex formation enhances the host IgG degradation [103]. Thus, we propose that in* T. vaginalis* iron could help in the selection of specific trichocystatin CP targets from the full range of expressed peptidases and that this protein-protein complex formation could modulate the appropriate biological effect, depending on the different locations where this interaction occurs.

#### 6.3.2. Posttranslational Modifications (PTMs) Modulated by Iron May Help to Regulate the Specific Function of Each Trichomonad CP

PTMs play crucial roles in regulating the diverse protein-protein interactions involved in essentially every cellular process and therefore are required in every microorganism for its development. To date, PTM characterization in* T. vaginalis* has been reported for only a few proteins, for example, TveIF5a, cytoskeletal proteins, tubulin, and several virulence factors, P270, AP120, and TvCP39 [7, 52, 57, 104].

*In silico* analysis of several CP-encoding genes predicted distinct types of PTMs, glycosylation (O- or N-glycosylation), and phosphorylation among others that were also suggested after analysis of the proteome reference map of* T. vaginalis* [31]. Furthermore, based on these analyses, it is proposed that* T. vaginalis* has the machinery to perform both O- and N-glycosylation of proteins [105]. Protein phosphorylation is undoubtedly the most common and best studied of PTMs and* T. vaginalis* has one of the largest eukaryotic kinomes known [25, 79], suggesting that this parasite may perform protein phosphorylation reactions under different environmental conditions and through different signaling pathways. Surprisingly,* T. vaginalis* lacks PK receptors-coding genes that facilitate the transduction of extracellular signals [25].

The cytotoxic TvCP39 is N-glycosylated and is highly immunogenic [7, 27, 52, 57]. TvCP39 is the first glycosylated CP detected in* T. vaginalis.* However, we still do not know whether glycosylation is necessary for TvCP39 activation or modulates its proteolytic activity or even its interaction with the endogenous inhibitor TC-2, nor whether this is also modulated by iron and could help to explain changes in its molecular size as detected by MS.

### 6.4. Other Possible Mechanisms for CP Gene Regulation through Gene Silencing

#### 6.4.1. By MicroRNAs

miRNAs are small, noncoding, double-stranded RNA found in many eukaryotic organisms that regulate different cellular process (proliferation, differentiation, apoptosis, and response to stress), modulating the mRNA translation efficiency, the mRNA degradation by binding to complementary sequences on the target mRNAs, and inducing posttranscriptional silencing. These types of RNAs because they are small interfering RNAs (siRNAs) activate the RNA interference machinery. These miRNA are transcribed by the RNApol II and processed into a 60-nucleotide precursor and exported from the nucleus to the cytoplasm by the exportin-5 and Ran-GTPase proteins. Cytosolic Dicer and Argonaute proteins process this precursor to a mature miRNA or siRNA.

In the* T. vaginalis* genome sequence there are Dicer- and Argonaute-encoding genes [25] as well as Exportin-5 and Ran-GTPase orthologues, and several miRNAs have been recently identified [106], suggesting that this parasite could employ these small RNAs transcribed from intergenic regions to regulate the expression of massively expanded gene families [25, 79]. These RNAs may play an important role in the regulation of several highly repeated gene families in the genome such as the cysteine proteinase families [76]. It is proposed that some genes that belong to multigene families could be transcribed and function as siRNAs. In these cases, the organisms could use the interference RNA machinery to modulate the expression of this type of multigene families. So far, this type of regulation has been little explored in trichomonads and it is unknown whether iron can influence this type of regulation. However, it could be an explanation for understanding how genes encoding some CPs are expressed at low mRNA level (seven in low-iron conditions, Tables 2 and 3) and no proteins have been found yet. Recently, Woehle et al. [107] demonstrated the expression of intergenic loci including numerous transcribed pseudogenes and long noncoding RNAs that can act as regulatory RNAs too.

#### 6.4.2. Repetitive Elements

As previously mentioned,* T. vaginalis* contains several repetitive elements in its genome. One of them is the* Tc1/mariner* transposable element (TE) superfamily (a type of DNA sequence that can change its position within the genome. It belongs to one of the most diverse and widespread class II TEs). Bradic et al. [108] investigated the abundance and distribution of a subset of 19* Tvmar1* loci in different* T. vaginalis* isolates. This research group determined the effect of* Tvmar1* insertion on the* T. vaginalis* gene expression and found that mRNA expression positively correlates with an increase in the distance of the* Tvmar1* locus for genes that have a* Tvmar1* insertion located in the 5′-upstream region.

The* in silico* analysis of the CP genomic organization reveals the presence of mariner elements close to the 5′-region in some CP genes like the untranscribed CP (TVAG_218830) [25]. This analysis also shows that some genes that do not have reported mRNAs possess mariner elements. In addition, the other genes belonging to the same contig or located nearby are not transcribed either. Interestingly, most of these genes contain several repeated elements located at both ends of the contig. Thus, it will be very interesting to explore the other possible regulatory mechanism of gene silencing in* T. vaginalis* as selective for CP gene expression that could be related to the iron concentrations to release or maintain this blockage as Bradic et al. [108] reported.

#### 6.4.3. Epigenetic Mechanisms

Another unexplored mechanism could be related to epigenetic factors that may control CP gene expression at the chromatin level. Chen et al. [109] demonstrate a novel DNA sequence periodicity signature of nucleosome organization in* T. vaginalis*, suggesting that nucleosomes present the right position and with regularity near to the 5′-end of transcripts. We conducted a search for potential chromatin-remodeling and histone-modifying proteins in the* T. vaginalis* genome database. We found the presence of several genes encoding histone acetylases and deacetylases. One of these genes (TVAG_319320) [25] is a member of the Sir2 family or sirtuins.

Sirtuins are NAD^+^-dependent protein N*ε*-acetyl-lysine (AcK) deacetylases that could also have mono-ADP-ribosyltransferase activity. Although Sir2 main function is as a histone deacetylase able to downregulate the transcription of their target genes by controlling chromatin structure and function, it is also capable of deacetylating other nuclear and cytoplasmic proteins due to their multiple localizations. Sirtuins show function diversification mainly in four areas: chromatin organization, metabolic regulation, cell survival in stress conditions, and cell differentiation and development. Interestingly, a growing body of evidence suggests that, in a significant number of these new functions, the main effect of sirtuins is exerted via a direct effect on chromatin [110, 111].

The trichomonad Sir2-encoding gene (TVAG_319320) contains MRE2-r element in its 5′-region and its expression is negatively regulated by iron at the transcript level [71]. Thus, this type of trichomonad regulatory enzyme could play a key role in gene silencing of several CP genes in response to iron levels by a still unknown epigenetic regulatory mechanism. Work is in progress to explore this possibility.

## 7. Conclusion and Perspectives

**Figure 10:**
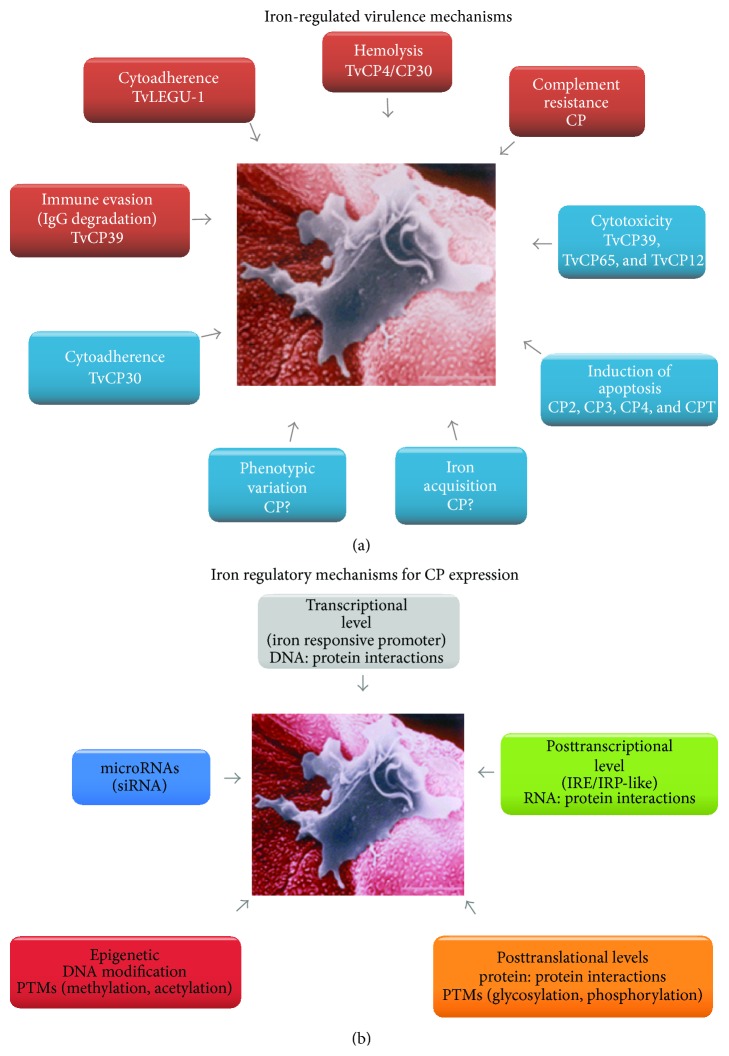
*T. vaginalis* virulence properties involving CPs and iron-depending mechanisms that regulate its expression. (a) Virulence mechanisms and CPs downregulated (blue) or upregulated (red) by iron. (b) Iron regulatory mechanisms implicated in CP gene expression at transcriptional, posttranscriptional, posttranslational, and epigenetic levels. Image modified from Arroyo et al. [112].

This report shows that iron plays a key role in the general physiology, morphology, and pathogenesis of* T. vaginalis*. This cation differentially modulates growth and virulence properties such as cytoadherence, cytotoxicity, hemolysis, induction of apoptosis in the host cell, complement resistance, and immune evasion, through induction or repression of the expression of cysteine proteinases as virulence factors (Figure 10).

Interestingly, the 220 CP-coding genes are grouped into different clans and most of them belong to multigene families. Multiple CP proteolytic activities are detected in 2D zymograms but corresponded to few different cathepsin L-like and legumain-like CPs whose mRNAs were also detected in the transcriptomic and EST analyses. These CP genes appear to be highly transcribed. Some of these CPs are differentially regulated by iron at transcriptional, posttranscriptional, or posttranslational levels. Herein, we offer some explanations supporting the selectivity in gene expression of some members of this multigene family that could be related to different virulence degrees, the type of isolate, the presence of TVV, or other unknown characteristics.

Some of the possible molecular mechanisms involved in gene expression regulation mediated by iron could be through (1)* DNA-protein interactions* by an iron responsive promoter including an MRE or MRE-like motif and Myb-like proteins, (2)* RNA-protein interactions* by atypical IRE hairpin mRNA structures and atypical cytoplasmic proteins causing translational blockage and mRNA stabilization in the absence of iron as occur with* tvcp4* or* tvcp12* expression, (3)* protein-protein interactions* between trichocystatin endogenous CP inhibitors and the target CPs as in TC-2 and TvCP39 interaction to control the unwanted proteolytic activity in the parasite, (4)* posttranslational modifications* such as phosphorylation and glycosylation as in TvCP39 and TvLEGU-1 that could have an important role in CP activation and immunogenicity in the host during infection. Transcriptional blockage (5) by* Tvmar-1 repetitive elements* (presence, number, and position) and (6) by* miRNAs* that are carried on the specific mRNA degradation through the interference machinery, and (7)* an epigenetic mechanism* that could also be involved in iron regulation possibly through the differential expression of a NAD^+^-dependent protein deacetylase* sir2-*encoding gene that could be expressed depending on the iron concentration and modulate gene expression by deacetylation of histones and other cytoplasmic regulatory proteins (Figure 10).

Thus, as shown herein, the transcriptomic and proteomic analysis in* T. vaginalis* is not enough to explain all the possible mechanisms involved in gene expression regulation of CPs mediated by iron due to the complexity observed for this early divergent protist, leaving many aspects of the parasite biology unexplained for future work to come in the following years.

## Figures and Tables

**Table 1:** Cysteine proteinases in virulence properties of *Trichomonas vaginalis*.

Proteinase	Virulence properties	Regulation by iron	Type of regulation	Ref.
CP (30 kDa)	Cytoskeleton disruption	ND	ND	[61]
TvCP4	Hemolysis	+	Posttranscriptional level	[36, 58]
TvCP12	Cytotoxicity	−	Posttranscriptional level	[7, 54, 57]
TvCP30	Cytoadherence Protein degradation	−	ND	[7, 52]
TvCP39	Cytotoxicity Igs Degradation	−	Posttranscriptional level Posttranslational level	[7, 52, 57]
TvCP62	Cytoadherence	+	ND	[52, 55]
TvCP65	Cytotoxicity	−	Transcriptional level Posttranscriptional level Posttranslational level	[7, 52, 56]
CP2, CP3, CP4, and CPT	Induction of host cell apoptosis	−	ND	[65, 66]
CP1	ND	ND	Transcriptional level	[27, 35, 71]
TvLEGU-1	Cytoadherence	+	Transcriptional level Posttranslational level	[7, 43, 52, 54]

ND: not determined; upregulated (+); downregulated (−).

**Table 2:** Expression at the mRNA and protein levels of cathepsin L-like and papain-like cysteine proteinases of *Trichomonas vaginalis*.

Type of CP	ID	Name	bp	aa	Contig	Orientation	RE	EST		T^b^	Exp. reports	Proteome	Ref.
	Accession number							Total	TvLI				
CB-like	**TVAG_488380** gb∣XP_001323959.1 gi∣123483120	NA	765	255	DS113314	3′-5′	4	0	0	NR	NR	NR	[25]

CL-like	**TVAG_485880** gb∣XP_001321164.1 gi∣123475979	NA	1356	452	DS113367	3′-5′	NP	10	1	NR	NR	NR	[25]

CL-like	**TVAG_484350** gb∣XP_001304351.1 gi123413805	NA^a^	873	291	DS114036	5′-3′	NP	9	2	NR	NR	NR	[25]

CB-like	**TVAG_482410** gb∣XP_001322190.1 gi∣123478051	NA	864	288	DS113347	3′-5′	NP	0	0	NR	NR	NR	[25]

CL-like	**TVAG_46797** gb∣XP_001326005.1^1^ gi∣123492185^1^ gb∣AAV98582^2^ gi∣56567186^2^ gb∣CAA54438^3^ gi∣454890^3^	TvCP4	915	305	DS113280	5′-3′	NP	215	19	NR	L/H	L	[7, 25, 27, 28, 35, 58, 65, 66, 71]

CL-like	**TVAG_465470** gb∣XP_001328382.1 gi∣123502829	NA^a^	915	305	DS113246	5′-3′	NP	0	0	NR	NR	NR	[25]

CL-like	**TVAG_461630** gb∣XP_001297865.1 gi∣123375585	NA	702	234	DS115136	3′-5′	NP	0	0	NR	NR	NR	[25]

CB-like	**TVAG_454200** gb∣XP_001329727.1 gi∣123508810	NA	759	253	DS113229	5′-3′	NP	0	0	NR	NR	NR	[25]

CL-like	**TVAG_437820** gb∣XP_001310334.1 gi∣123439119	NA^a^	915	305	DS113716	5′-3′	2	0	0	NR	NR	NR	[25]

CL-like	**TVAG_427120** gb∣XP_001306414.1 gi∣123423610	NA	405	135	DS113910	5′-3′	NP	0	0	NR	NR	NR	[25]

CL-like	**TVAG_427130** gb∣XP_001306415.1 gi∣123423613	NA	399	133	DS113910	5′-3′	NP	4	0	NR	NR	NR	[25]

CL-like	**TVAG_410260** gb∣XP_001323249.1 gi∣123480189	TvCP12	945	315	DS113327	5′-3′	NP	29	3	NR	L	NR	[25]

CL-like	**TVAG_405280** gb∣XP_001325205.1 gi∣123488591	NA^a^	918	306	DS113293	3′-5′	NP	0	0	NR	NR	NR	[25]

CL-like	**TVAG_398510** gb∣XP_001304940.1 gi∣123416650	NA	411	137	DS113996	3′-5′	3	0	0	NR	NR	NR	[25]

CL-like	**TVAG_355480** gb∣XP_001310117.1 gi∣123438675	TvCP4-like	915	305	DS113726	5′-3′	1	0	0	NR	NR	NR	[25, 27]

CL-like	**TVAG_328620** gb∣XP_001313029.1 gi∣123448602	NA	303	101	DS113614	3′-5′	NP	0	0	NR	NR	NR	[25]

CL-like	**TVAG_325040** gb∣XP_001330238.1 gi∣123975201	NA^a^	873	291	DS113569	5′-3′	NP	5	1	NR	NR	NR	[25]

CL-like	**TVAG_302570** gb∣XP_001320393.1 gi∣123474420	NA	1479	493	DS113384	5′-3′	NP	3	0	NR	NR	NR	[25]

CL-like	**TVAG_298080** gb∣XP_001316414.1^1^ gi∣123457373^1^ gb∣ABX56032.1^4^ gi∣161016200^4^	TvCPT/ TvCP39	915	305	DS113482	3′-5′	1	168	8	L	L	N	[25, 27–29, 57, 65, 66]

CL-like	**TVAG_293170** gb∣XP_001298580.1 gi∣123381478	NA	399	133	DS114872	5′-3′	3	0	0	NR	NR	NR	[25]

CL- or K-like	**TVAG_267850** gb∣XP_001579738.1 gi∣154413416	NA^a^	960	320	DS113215	5′-3′	NP	0	0	NR	NR	NR	[25]

CL-like	**TVAG_257790** gb∣XP_001313154.1 gi∣123448858	NA	609	203	DS113609	3′-5′	NP	1	0	NR	NR	NR	[25]

CL-, S-, or H-like	**TVAG_242850** gb∣XP_001311850.1 gi∣123446194	NA	1419	473	DS113657	5′-3′	NP	5	1	NR	NR	NR	[25]

CL-like	**TVAG_228710** gb∣XP_001580594.1 gi∣154415137	NA	879	293	DS113206	5′-3′	NP	1	0	NR	NR	NR	[25]

CL-like	**TVAG_228450** gb∣XP_001580568.1 gi∣154415085	NA^a^	915	305	DS113206	5′-3′	1	0	0	NR	NR	NR	[25]

CL-like	**TVAG_218830** gb∣XP_001300036.1 gi∣123391254	NA	399	133	DS114510	3′-5′	NP	6	0	NR	NR	NR	[25]

CB-like	**TVAG_216350** gb∣XP_001317882.1 gi∣123469339	NA	723	241	DS113443	5′-3′	NP	0	0	NR	NR	NR	[25]

CL-like	**TVAG_202090** gb∣XP_001327438.1^1^ gi∣123498602^1^ gb∣CAA54435.1^3^ gi∣452292^3^	CP1	927	309	DS113259	3′-5′	NP	69	12	L	L	N	[25, 27–29, 35, 65, 66, 71]

CH-like	**TVAG_181310** XP_001306268.1 GI:123422894	NA	1464	488	DS113918	5′-3′	NP	0	1	NR	NR	NR	[25]

CL-like	**TVAG_164120** gb∣XP_001325526.1 giI123490067	NA^a^	915	305	DS113287	5′-3′	1	4	1	NR	NR	NR	[25]

CB-like	**TVAG_159150** gb∣XP_001298125.1 gi∣123377855	NA	405	135	DS115034	3′-5′	NP	0	0	NR	NR	NR	[25]

CL- or K-like	**TVAG_151460** gb∣XP_001300085.1 gi∣123391522	TvCP25	855	285	DS114500	5′-3′	NP	7	0	NR	NR	NR	[25, 33]

CL-like	**TVAG_146680** gb∣XP_001579911.1 gi∣154413764	NA	951	317	DS113213	5′-3′	1	2	0	NR	NR	NR	[25]

CL-like	**TVAG_135480** gb∣XP_001290980.1 gi∣123299807	NA^a^	792	264	DS122112	3′-5′	NP	1	1	NR	NR	NR	[25]

CL- or H-like	**TVAG_126090** gb∣XP_001321671.1 gi∣123477003	NA	1305	435	DS113357	5′-3′	NP	5	3	NR	NR	NR	[25]

P-like	**TVAG_096740** gb∣XP_001313692.1 gi∣123449986	CP65^a^	915	305	DS113590	5′-3′	NP	0	0	NR	NR	NR	[25]

P-like	**TVAG_090100** gb∣XP_001314419.1^1^ gi∣123976011^1^ gb∣CAA54437.1^3^ gi∣452296^3^	CP3^a^	954	318	DS113554	3′-5′	NP	178	9	L	L	L	[25, 27, 28, 35, 65, 66]

P-like	**TVAG_057000** gb∣XP_001319129.1^1^ gi∣123471864^1^ gb∣CAA54436.1^3^ gi∣452294^3^	TvCP2^a^	942	314	DS113412	3′-5′	3	198	56	L	L	N	[25, 27, 31, 35, 65, 66]

P-like	**TVAG_053440** gb∣XP_001318243.1 gi∣123470070	NA^a^	882	294	DS113434	5′-3′	NP	1	0	NR	NR	NR	[25]

P-like	**TVAG_052570** gb∣XP_001308929.1 gi∣123435098	NA	300	100	DS113779	3′-5′	NP	0	0	NR	NR	NR	[25]

P-like	**TVAG_047960** gb∣XP_001330457.1 gi∣123976147	NA^a^	900	300	DS113552	3′-5′	NP	2	0	NR	NR	NR	[25]

P-like	**TVAG_043620** gb∣XP_001315639.1 giI123455797	NA^a^	915	305	DS113505	5′-3′	NP	0	0	NR	NR	NR	[25]

P-like	**TVAG_040870** gb∣XP_001315160.1 gi∣123454821	NA	1386	462	DS113520	3′-5′	NP	8	2	NR	NR	NR	[25]

P-like	**TVAG_034140** gb∣XP_001318458.1 gi∣123470506	NA^a^	951	317	DS113428	3′-5′	NP	20	0	NR	NR	NR	[25]

P-like	**TVAG_028720** gb∣XP_001326142.1 gi∣123492781	NA	723	241	DS113278	3′-5′	9	0	0	NR	NR	NR	[25]

P-like	**TVAG_003650** gb∣XP_001324382.1 gi∣123484966	NA^a^	930	310	DS113306	3′-5′	NP	1	0	NR	NR	NR	[25]

P-like	**TVAG_518810** gb∣XP_001283092.1 gi∣123194565	NA^a^	942	314	DS136773	5′-3′	NP	7	1	NR	NR	NR	[25]

CB-like: cathepsin B-like CP; CL-like: cathepsin L-like CP; P-like: papain-like CP. ID: gene identification in the TrichDB (http://www.trichdb.org/) genome database or in PubMed database; NA: nonassigned name; bp: gene size in base pairs (bp); aa: protein size in amino acids (aa); contig: contig number identification; orientation: gene orientation in the contig; RE: number of repetitive elements close to CP localized in the same contig; NP: not present; EST and TvLI: Total of EST sequence and ESTs reported in low-iron conditions in TrichDB database, respectively; NR: nonreported data; H: high-iron conditions; N: normal iron conditions: L: low-iron conditions: T: upregulated genes in H or L iron concentrations.

^a^Proteins presenting certain homology degree with TvCP4; ^b^transcriptome information by Horváthová et al., 2012 [71]. ^1^Accession number reported by Carlton et al., 2007 [25]; ^2^accession number reported by Solano-González et al., 2007 [36]; ^3^accession number reported by Mallinson et al., 1994 [35]; ^4^accession number reported by Sommer et al., 2005 [65].

**Table 3:** Expression at the mRNA and protein levels of legumain-like cysteine proteinases of *Trichomonas vaginalis*.

Type of CP	ID	Name	bp	aa	Contig	Orientation	RE	Total		T^a^	Exp. reports	Proteome	Ref.
	Accession number							EST	TvLI				
AEP-like	**TVAG_426660** gb∣XP_001326695.1 gi∣123495228^1^ gb∣AAQ93039.1^2^ gi∣39573850^2^	TvLEGU-1	1164	388	DS113270	3′-5′	NP	177	10	H	H	ID	[25, 27, 30, 42, 43, 71]

AEP-like	**TVAG_385340** gb∣XP_001303267.1 gi∣123408789	TvLEGU-2	1176	392	DS114117	3′-5′	1	33	1	NR	NR	N	[25, 29, 42]

AEP-like	**TVAG_328450** gb∣XP_001313012.1 gi∣123448568	TvLEGU-4	1176	392	DS113614	3′-5′	8	1	0	NR	NR	NR	[25]

AEP-like	**TVAG_305110** gb∣XP_001299781.1 gi∣123389835	TvLEGU-9	1245	415	DS114558	3′-5′	3	6	2	NR	NR	NR	[25]

AEP-like	**TVAG_277470** gb∣XP_001304607.1 gi∣123415014	TvLEGU-10	1140	380	DS114018	3′-5′	1	0	0	NR	NR	NR	[25]

AEP-like	**TVAG_185540** gb∣XP_001584233.1 gi∣154422442	TvLEGU-6	1134	378	DS113179	3′-5′	NP	4	0	NR	NR	NR	[25]

AEP-like	**TVAG_068410** gb∣XP_001307303.1 gi∣123427668	TvLEGU-8	1134	378	DS113861	3′-5′	NP	1	0	NR	NR	NR	[25]

AEP-like	**TVAG_060430** gb∣XP_001321890.1 gi∣123477445	TvLEGU-5	1179	393	DS113353	3′-5′	NP	0	0	NR	NR	NR	[25]

AEP-like	**TVAG_050390** gb∣XP_001319446.1 gi∣123472505	TvLEGU-3	1215	405	DS113405	5′-3′	1	13	1	NR	NR	NR	[25]

AEP-like	**TVAG_035520** gb∣XP_001583515.1 gi∣154421002	TvLEGU-7	1134	378	DS113183	5′-3′	1	0	0	NR	NR	NR	[25]

AEP-like: asparaginyl endopeptidase-like; ID: gene identification in TrichDB genome database [25] or in PubMed database; bp: gene size in base pairs (bp); aa: protein size in amino acids (aa); contig: contig number identification; orientation: gene orientation in the contig; RE: number of repetitive elements close to CP localized in the same contig; NP: not present RE; NR: no reported data; EST and TvLI: total of EST sequence and ESTs reported in low-iron conditions in the TrichDB database, respectively; H: high-iron conditions; T^a^: upregulated genes in H or L iron concentrations by transcriptomic analysis [71].

## References

[1] Kühn L. C. (2009). How iron controls iron. *Cell Metabolism*.

[2] Testa U. (2000). *Proteins of Iron Metabolism*.

[3] Pantopoulos K. (2004). Iron metabolism and the IRE/IRP regulatory system: an update. *Annals of the New York Academy of Sciences*.

[4] Weinberg E. D. (1974). Iron and susceptibility to infectious disease. *Science*.

[5] Schwebke J. R., Burgess D. (2004). Trichomoniasis. *Clinical Microbiology Reviews*.

[6] Lehker M. W., Alderete J. F. (2000). Biology of trichomonosis. *Current Opinion in Infectious Diseases*.

[7] Figueroa-Angulo E. E., Rendón-Gandarilla F. J., Puente-Rivera J. (2012). The effects of environmental factors on the virulence of *Trichomonas vaginalis*. *Microbes and Infection*.

[8] Conrad M., Zubacova Z., Dunn L. A. (2011). Microsatellite polymorphism in the sexually transmitted human pathogen *Trichomonas vaginalis* indicates a genetically diverse parasite. *Molecular and Biochemical Parasitology*.

[9] Conrad M. D., Gorman A. W., Schillinger J. A. (2012). Extensive genetic diversity, unique population structure and evidence of genetic exchange in the sexually transmitted parasite *Trichomonas vaginalis*. *PLoS Neglected Tropical Diseases*.

[10] Provenzano D., Khoshnan A., Alderete J. F. (1997). Involvement of dsRNA virus in the protein composition and growth kinetics of host *Trichomonas vaginalis*. *Archives of Virology*.

[11] Ryan C. M., de Miguel N., Johnson P. J. (2011). *Trichomonas vaginalis*: current understanding of host-parasite interactions. *Essays in Biochemistry*.

[12] Hirt R. P., de Miguel N., Nakjang S. (2011). *Trichomonas vaginalis* pathobiology new insights from the genome sequence. *Advances in Parasitology*.

[13] Gorrell T. E. (1985). Effect of culture medium iron content on the biochemical composition and metabolism of *Trichomonas vaginalis*. *Journal of Bacteriology*.

[14] Lehker M. W., Alderete J. F. (1992). Iron regulates growth of *Trichomonas vaginalis* and the expression of immunogenic trichomonad proteins. *Molecular Microbiology*.

[15] Lehker M. W., Chang T. H., Dailey D. C., Alderete J. F. (1990). Specific erythrocyte binding is an additional nutrient acquisition system for *Trichomonas vaginalis*. *The Journal of Experimental Medicine*.

[16] Lehker M. W., Arroyo R., Alderete J. F. (1991). The regulation by iron of the synthesis of adhesins and cytoadherence levels in the protozoan *Trichomonas vaginalis*. *Journal of Experimental Medicine*.

[17] Ardalan S., Craig Lee B., Garber G. E. (2009). *Trichomonas vaginalis*: the adhesins AP51 and AP65 bind heme and hemoglobin. *Experimental Parasitology*.

[18] Granger B. L., Warwood S. J., Benchimol M., De Souza W. (2000). Transient invagination of flagella by *Tritrichomonas foetus*. *Parasitology Research*.

[19] Pereira-Neves A., Ribeiro K. C., Benchimol M. (2003). Pseudocysts in trichomonads—new insights. *Protist*.

[20] Benchimol M. (2004). Trichomonads under microscopy. *Microscopy and Microanalysis*.

[21] Jesus J. B., Vannier-Santos M. A., Britto C. (2004). *Trichomonas vaginalis* virulence against epithelial cells and morphological variability: the comparison between a well-established strain and a fresh isolate. *Parasitology Research*.

[22] de Jesus J. B., Cuervo P., Junqueira M. (2007). Application of two-dimensional electrophoresis and matrix-assisted laser desorption/ionization time-of-flight mass spectrometry for proteomic analysis of the sexually transmitted parasite Trichomonas vaginalis. *Journal of Mass Spectrometry*.

[23] Sajid M., McKerrow J. H. (2002). Cysteine proteases of parasitic organisms. *Molecular and Biochemical Parasitology*.

[24] Rawlings N. D., Waller M., Barrett A. J., Bateman A. (2014). MEROPS: the database of proteolytic enzymes, their substrates and inhibitors. *Nucleic Acids Research*.

[25] Carlton J. M., Hirt R. P., Silva J. C. (2007). Draft genome sequence of the sexually transmitted pathogen *Trichomonas vaginalis*. *Science*.

[26] Neale K. A., Alderete J. F. (1990). Analysis of the proteinases of representative *Trichomonas vaginalis* isolates. *Infection and Immunity*.

[27] Ramón-Luing L. A., Rendón-Gandarilla F. J., Cárdenas-Guerra R. E. (2010). Immunoproteomics of the active degradome to identify biomarkers for *Trichomonas vaginalis*. *Proteomics*.

[28] Cuervo P., Cupolillo E., Britto C. (2008). Differential soluble protein expression between *Trichomonas vaginalis* isolates exhibiting low and high virulence phenotypes. *Journal of Proteomics*.

[29] De Jesus J. B., Cuervo P., Britto C. (2009). Cysteine peptidase expression in *Trichomonas vaginalis* isolates displaying High- and low-virulence phenotypes. *Journal of Proteome Research*.

[30] de Jesus J. B., Cuervo P., Junqueira M. (2007). A further proteomic study on the effect of iron in the human pathogen *Trichomonas vaginalis*. *Proteomics*.

[31] Huang K.-Y., Chien K.-Y., Lin Y.-C. (2009). A proteome reference map of *Trichomonas vaginalis*. *Parasitology Research*.

[32] Polgár L., Halász P. (1982). Current problems in mechanistic studies of serine and cysteine proteinases. *Biochemical Journal*.

[33] León-Sicairos C. R., León-Félix J., Arroyo R. (2004). Tvcp12: a novel *Trichomonas vaginalis* cathepsin L-like cysteine proteinase-encoding gene. *Microbiology*.

[34] Hernandez-Gutierrez R., Ortega-López J., Arroyo R. (2003). A 39-kDa cysteine proteinase CP39 from *Trichomonas vaginalis*, which is negatively affected by iron may be involved in trichomonal cytotoxicity. *The Journal of Eukaryotic Microbiology*.

[35] Mallinson D. J., Lockwood B. C., Coombs G. H., North M. J. (1994). Identification and molecular cloning of four cysteine proteinase genes from the pathogenic protozoon *Trichomonas vaginalis*. *Microbiology*.

[36] Solano-González E., Burrola-Barraza E., León-Sicairos C. (2007). The trichomonad cysteine proteinase TVCP4 transcript contains an iron-responsive element. *FEBS Letters*.

[37] Lecaille F., Kaleta J., Brömme D. (2002). Human and parasitic papain-like cysteine proteases: their role in physiology and pathology and recent developments in inhibitor design. *Chemical Reviews*.

[38] Cárdenas-Guerra R. E., Ortega-López J., Flores-Pucheta C. I., Benítez-Cardoza C. G., Arroyo R. (2015). The recombinant prepro region of TvCP4 is an inhibitor of cathepsin L-like cysteine proteinases of *Trichomonas vaginalis* that inhibits trichomonal haemolysis. *The International Journal of Biochemistry & Cell Biology*.

[39] Roy A., Kucukural A., Zhang Y. (2010). I-TASSER: a unified platform for automated protein structure and function prediction. *Nature Protocols*.

[40] Roy A., Yang J., Zhang Y. (2012). COFACTOR: an accurate comparative algorithm for structure-based protein function annotation. *Nucleic Acids Research*.

[41] Zhang Y. (2008). I-Tasser server for protein 3D structure prediction. *BMC Bioinformatics*.

[42] León-Félix J., Ortega-López J., Orozco-Solís R., Arroyo R. (2004). Two novel asparaginyl endopeptidase-like cysteine proteinases from the protist *Trichomonas vaginalis*: their evolutionary relationship within the clan CD cysteine proteinases. *Gene*.

[43] Rendón-Gandarilla F. J., de los Angeles Ramón-Luing L., Ortega-López J., de Andrade I. R., Benchimol M., Arroyo R. (2013). The TvLEGU-1, a legumain-like cysteine proteinase, plays a key role in *Trichomonas vaginalis* cytoadherence. *BioMed Research International*.

[44] Chen J. M., Dando P. M., Rawlings N. D. (1997). Cloning, isolation, and characterization of mammalian legumain, an asparaginyl endopeptidase. *The Journal of Biological Chemistry*.

[45] Chen J.-M., Fortunato M., Barrett A. J. (2000). Activation of human prolegumain by cleavage at a C-terminal asparagine residue. *Biochemical Journal*.

[46] Dall E., Brandstetter H. (2013). Mechanistic and structural studies on legumain explain its zymogenicity, distinct activation pathways, and regulation. *Proceedings of the National Academy of Sciences of the United States of America*.

[47] Dall E., Brandstetter H. (2012). Activation of legumain involves proteolytic and conformational events, resulting in a context-and substrate-dependent activity profile. *Acta Crystallographica Section F: Structural Biology and Crystallization Communications*.

[48] Mottram J. C., Helms M. J., Coombs G. H., Sajid M. (2003). Clan CD cysteine peptidases of parasitic protozoa. *Trends in Parasitology*.

[49] Asgian J. L., James K. E., Li Z. Z. (2002). Aza-peptide epoxides: a new class of inhibitors selective for clan CD cysteine proteases. *Journal of Medicinal Chemistry*.

[50] Zhao L., Hua T., Crowley C. (2014). Structural analysis of asparaginyl endopeptidase reveals the activation mechanism and a reversible intermediate maturation stage. *Cell Research*.

[51] Ryu J. S., Choi H. K., Min D. Y., Ha S. E., Ahn M. H. (2001). Effect of iron on the virulence of *Trichomonas vaginalis*. *Journal of Parasitology*.

[52] Hernández H. M., Marcet R., Sarracent J. (2014). Biological roles of cysteine proteinases in the pathogenesis of. *Parasite*.

[53] Garber G. E., Lemchuk-Favel L. T., Bowie W. R. (1989). Isolation of a cell-detaching factor of *Trichomonas vaginalis*. *Journal of Clinical Microbiology*.

[54] Torres-Romero J. C., Arroyo R. (2009). Responsiveness of *Trichomonas vaginalis* to iron concentrations: evidence for a post-transcriptional iron regulation by an IRE/IRP-like system. *Infection, Genetics and Evolution*.

[55] Hernández H., Sariego I., Garber G., Delgado R., López O., Sarracent J. (2004). Monoclonal antibodies against a 62 kDa proteinase of *Trichomonas vaginalis* decrease parasite cytoadherence to epithelial cells and confer protection in mice. *Parasite Immunology*.

[56] Alvarez-Sánchez M. E., Solano-González E., Yañez-Gómez C., Arroyo R. (2007). Negative iron regulation of the CP65 cysteine proteinase cytotoxicity in *Trichomonas vaginalis*. *Microbes and Infection*.

[57] Ramón-Luing L. D. L. Á., Rendón-Gandarilla F. J., Puente-Rivera J., Ávila-González L., Arroyo R. (2011). Identification and characterization of the immunogenic cytotoxic TvCP39 proteinase gene of *Trichomonas vaginalis*. *The International Journal of Biochemistry & Cell Biology*.

[58] Cárdenas-Guerra R. E., Arroyo R., Rosa de Andrade I., Benchimol M., Ortega-López J. (2013). The iron-induced cysteine proteinase TvCP4 plays a key role in *Trichomonas vaginalis* haemolysis. *Microbes and Infection*.

[59] Dailey D. C., Chang T.-H., Alderete J. F. (1990). Characterization of *Trichomonas vaginalis* haemolysis. *Parasitology*.

[60] Fiori P. L., Rappelli P., Addis M. F., Mannu F., Cappuccinelli P. (1997). Contact-dependent disruption of the host cell membrane skeleton induced by *Trichomonas vaginalis*. *Infection and Immunity*.

[61] Fiori P. L., Rappelli P., Addis M. F. (1999). The flagellated parasite *Trichomonas vaginalis*: new insights into cytopathogenicity mechanisms. *Microbes and Infection*.

[62] Alderete J. F., Provenzano D., Lehker M. W. (1995). Iron mediates *Trichomonas vaginalis* resistance to complement lysis. *Microbial Pathogenesis*.

[63] Provenzano D., Alderete J. F. (1995). Analysis of human immunoglobulin-degrading cysteine proteinases of *Trichomonas vaginalis*. *Infection and Immunity*.

[64] Chang J.-H., Ryang Y.-S., Kim S.-K., Park J.-Y. (2004). *Trichomonas vaginalis*-induced apoptosis in RAW264.7 cells is regulated through Bcl-x_l_, but not Bcl-2. *Parasite Immunology*.

[65] Sommer U., Costello C. E., Hayes G. R. (2005). Identification of *Trichomonas vaginalis* cysteine proteases that induce apoptosis in human vaginal epithelial cells. *The Journal of Biological Chemistry*.

[66] Kummer S., Hayes G. R., Gilbert R. O., Beach D. H., Lucas J. J., Singh B. N. (2008). Induction of human host cell apoptosis by *Trichomonas vaginalis* cysteine proteases is modulated by parasite exposure to iron. *Microbial Pathogenesis*.

[67] Hirt R. P. (2013). *Trichomonas vaginalis* virulence factors: an integrative overview. *Sexually Transmitted Infections*.

[68] Alderete J. F., Newton E., Dennis C., Neale K. A. (1991). The vagina of women infected with *Trichomonas vaginalis* has numerous proteinases and antibody to trichomonad proteinases. *Genitourinary Medicine*.

[69] Alderete J. F., Newton E., Dennis C., Neale K. A. (1991). Antibody in sera of patients infected with *Trichomonas vaginalis* is to trichomonad proteinases. *Genitourinary Medicine*.

[70] Alderete J. F., Provenzano D. (1997). The vagina has reducing environment sufficient for activation of *Trichomonas vaginalis* cysteine proteinases. *Genitourinary Medicine*.

[71] Horváthová L., Šafaříková L., Basler M. (2012). Transcriptomic identification of iron-regulated and iron-independent gene copies within the heavily duplicated *Trichomonas vaginalis* genome. *Genome Biology and Evolution*.

[72] Coombs G. H., North M. J. (1983). An analysis of the proteinases of *Trichomonas vaginalis* by polyacrylamide gel electrophoresis. *Parasitology*.

[73] North M. J., Robertson C. D., Coombs G. H. (1990). The specificity of trichomonad cysteine proteinases analysed using fluorogenic substrates and specific inhibitors. *Molecular and Biochemical Parasitology*.

[74] Lockwood B. C., North M. J., Scott K. I., Bremner A. F., Coombs G. H. (1987). The use of a highly sensitive electrophoretic method to compare the proteinases of trichomonads. *Molecular and Biochemical Parasitology*.

[75] Huang K.-Y., Huang P.-J., Ku F.-M., Lin R., Alderete J. F., Tanga P. (2012). Comparative transcriptomic and proteomic analyses of *Trichomonas vaginalis* following adherence to fibronectin. *Infection and Immunity*.

[76] Jia W.-Z., Li Z., Zhao L., Lun Z. R. (2008). Genetic variation and clustal analysis of *Trichomonas vaginalis* cysteine proteases. *Chinese Journal of Parasitology & Parasitic Diseases*.

[77] Saska I., Craik D. J. (2008). Protease-catalysed protein splicing: a new post-translational modification?. *Trends in Biochemical Sciences*.

[78] Carvajal-Gamez B. I., Quintas-Granados L. I., Arroyo R. (2014). Putrescine-dependent re-localization of TvCP39, a cysteine proteinase involved in *Trichomonas vaginalis* cytotoxicity. *PLoS ONE*.

[79] Carlton J. M., Malik S.-B., Sullivan S. A., Sicheritz-Pontén T., Tang P., Hirt R. P. (2010). The genome of *Trichomonas vaginalis*. *Anaerobic Parasitic Protozoa: Genomics and Molecular Biology*.

[80] Liston D. R., Johnson P. J. (1999). Analysis of a ubiquitous promoter element in a primitive eukaryote: early evolution of the initiator element. *Molecular and Cellular Biology*.

[81] Smith A., Johnson P. (2011). Gene expression in the unicellular eukaryote *Trichomonas vaginalis*. *Research in Microbiology*.

[82] Smith A. J., Chudnovsky L., Simoes-Barbosa A. (2011). Novel core promoter elements and a cognate transcription factor in the divergent unicellular eukaryote *Trichomonas vaginalis*. *Molecular and Cellular Biology*.

[83] Liston D. R., Lau A. O. T., Ortiz D., Smale S. T., Johnson P. J. (2001). Initiator recognition in a primitive eukaryote: IBP39, an initiator-binding protein from *Trichomonas vaginalis*. *Molecular and Cellular Biology*.

[84] Schumacher M. A., Lau A. O. T., Johnson P. J. (2003). Structural basis of core promoter recognition in a primitive eukaryote. *Cell*.

[85] Tsai C.-D., Liu H.-W., Tai J.-H. (2002). Characterization of an iron-responsive promoter in the protozoan pathogen *Trichomonas vaginalis*. *Journal of Biological Chemistry*.

[86] Ong S.-J., Hsu H.-M., Liu H.-W., Chu C.-H., Tai J.-H. (2007). Activation of multifarious transcription of an adhesion protein ap65-1 gene by a novel Myb2 protein in the protozoan parasite *Trichomonas vaginalis*. *The Journal of Biological Chemistry*.

[87] Hsu H.-M., Ong S.-J., Lee M.-C., Tai J.-H. (2009). Transcriptional regulation of an iron-inducible gene by differential and alternate promoter entries of multiple Myb proteins in the protozoan parasite *Trichomonas vaginalis*. *Eukaryotic Cell*.

[88] Hsu H. M., Lee Y., Indra D. (2012). Iron-inducible nuclear translocation of a Myb3 transcription factor in the protozoan parasite *Trichomonas vaginalis*. *Eukaryotic Cell*.

[89] Hsu H., Lee Y., Hsu P. (2014). Signal transduction triggered by iron to induce the nuclear importation of a myb3 transcription factor in the parasitic protozoan *Trichomonas vaginalis*. *Journal of Biological Chemistry*.

[90] Puente-Rivera J., de los Ángeles Ramón-Luing L., Figueroa-Angulo E. E., Ortega-López J., Arroyo R. (2014). Trichocystatin-2 (TC-2): an endogenous inhibitor of cysteine proteinases in *Trichomonas vaginalis* is associated with TvCP39. *The International Journal of Biochemistry & Cell Biology*.

[91] Wang J., Pantopoulos K. (2011). Regulation of cellular iron metabolism. *Biochemical Journal*.

[92] Calla-Choque J. S., Figueroa-Angulo E. E., Ávila-González L., Arroyo R. (2014). *α*-actinin TvACTN3 of *Trichomonas vaginalis* is an RNA-binding protein that could participate in its posttranscriptional iron regulatory mechanism. *BioMed Research International*.

[93] Turk V., Stoka V., Turk D. (2008). Cystatins: biochemical and structural properties, and medical relevance. *Frontiers in Bioscience*.

[94] Rawlings N. D., Barrett A. J., Bateman A. (2012). MEROPS: the database of proteolytic enzymes, their substrates and inhibitors. *Nucleic Acids Research*.

[95] Abrahamson M., Barrett A. J., Salvesen G., Grubb A. (1986). Isolation of six cysteine proteinase inhibitors from human urine. Their physicochemical and enzyme kinetic properties and concentrations in biological fluids. *Journal of Biological Chemistry*.

[96] Abrahamson M., Alvarez-Fernandez M., Nathanson C.-M. (2003). Cystatins. *Biochemical Society Symposium*.

[97] Lalmanach G., Naudin C., Lecaille F., Fritz H. (2010). Kininogens: more than cysteine protease inhibitors and kinin precursors. *Biochimie*.

[98] Bode W., Engh R., Musil D. (1988). The 2.0 A X-ray crystal structure of chicken egg white cystatin and its possible mode of interaction with cysteine proteinases. *The EMBO Journal*.

[99] Turk B., Turk D., Salvesen G. S. (2002). Regulating cysteine protease activity: essential role of protease inhibitors as guardians and regulators. *Current Pharmaceutical Design*.

[100] Sanderson S. J., Westrop G. D., Scharfstein J., Mottram J. C., Coombs G. H. (2003). Functional conservation of a natural cysteine peptidase inhibitor in protozoan and bacterial pathogens. *FEBS Letters*.

[101] Santamaría M. E., Hernández-Crespo P., Ortego F. (2012). Cysteine peptidases and their inhibitors in *Tetranychus urticae*: a comparative genomic approach. *BMC Genomics*.

[102] Lee J.-Y., Song S.-M., Moon E.-K. (2013). Cysteine protease inhibitor (AcStefin) is required for complete cyst formation of Acanthamoeba. *Eukaryotic Cell*.

[103] Vincents B., Vindebro R., Abrahamson M., von Pawel-Rammingen U. (2008). The human protease inhibitor cystatin C is an activating cofactor for the streptococcal cysteine protease ides. *Chemistry and Biology*.

[104] Alderete J. F. (1999). Iron modulates phenotypic variation and phosphorylation of P270 in double-stranded RNA virus-infected *Trichomonas vaginalis*. *Infection and Immunity*.

[105] Paschinger K., Hykollari A., Razzazi-Fazeli E. (2012). The *N*-glycans of *Trichomonas vaginalis* contain variable core and antennal modifications. *Glycobiology*.

[106] Lin W.-C., Li S.-C., Shin J.-W. (2009). Identification of microRNA in the protist *Trichomonas vaginalis*. *Genomics*.

[107] Woehle C., Kusdian G., Radine C., Graur D., Landan G., Gould S. B. (2014). The parasite *Trichomonas vaginalis* expresses thousands of pseudogenes and long non-coding RNAs independently from functional neighbouring genes. *BMC Genomics*.

[108] Bradic M., Warring S. D., Low V., Carlton J. M. (2014). The Tc1/mariner transposable element family shapes genetic variation and gene expression in the protist *Trichomonas vaginalis*. *Mobile DNA*.

[109] Chen K., Meng Q., Ma L. (2008). A novel DNA sequence periodicity decodes nucleosome positioning. *Nucleic Acids Research*.

[110] Vaquero A. (2009). The conserved role of sirtuins in chromatin regulation. *The International Journal of Developmental Biology*.

[111] Silva J. P., Wahlestedt C. (2010). Role of Sirtuin 1 in metabolic regulation. *Drug Discovery Today*.

[112] Arroyo R., González-Robles A., Martínez-Palomo A., Alderete J. F. (1993). Signalling of *Trichomonas vaginalis* for amoeboid transformation and adhesin synthesis follows cytoadherence. *Molecular Microbiology*.

